# Co-occurring leukocytosis and hyperglycemia identify patients at high risk of subsequent mortality after intracerebral hemorrhage: a 24-h landmark analysis in MIMIC-IV and eICU

**DOI:** 10.3389/fneur.2026.1956229

**Published:** 2026-10-30

**Authors:** Hanrong Liang, Wenjuan Jing, Laixin Song, Diqun Wu, Shenhui Xiao, Haoxin Liu, Yuan Zhong

**Affiliations:** 1 Interventional Operating Room, School of Medicine, The Sixth Affiliated Hospital of South China University of Technology (Nanhai District People's Hospital of Foshan), Foshan, China; 2 Foshan Sanshui District People's Hospital, Foshan, Guangdong, China; 3 Department of Cerebrovascular Intervention, School of Medicine, The Sixth Affiliated Hospital of South China University of Technology (Nanhai District People's Hospital of Foshan), Foshan, China; 4 Department of Neurosurgery, Zhongnan Hospital of Wuhan University, Wuhan, China

**Keywords:** eICU, hyperglycemia, intracerebral hemorrhage, landmark analysis, leukocytosis, MIMIC-IV, mortality, risk stratification

## Abstract

**Background:**

White blood cell (WBC) count and glucose levels may jointly reflect physiological stress after intracerebral hemorrhage (ICH). We examined their associations with subsequent in-hospital mortality and whether they improved prediction beyond established clinical information.

**Methods:**

This retrospective study used the Medical Information Mart for Intensive Care IV (MIMIC-IV) and the eICU Collaborative Research Database. Adults with ICH identified by diagnosis codes who were alive and hospitalized 24 h after intensive care unit admission were included. WBC > 10 × 10^9^/L and glucose >140 mg/dL defined four groups: neither elevated, WBC elevation only, glucose elevation only, and both elevated. Logistic regression estimated adjusted associations, with bias reduction in the eICU cohort. Restricted cubic splines assessed nonlinear associations. Prediction models used the minimum first-day Glasgow Coma Scale score alone or a clinical baseline model that also included age, sex, blood pressure, and other laboratory measurements. Models were developed on MIMIC-IV and evaluated without updating in the eICU database.

**Results:**

The MIMIC-IV and eICU landmark cohorts included 1,606 and 264 patients, with 332 and 42 subsequent deaths, respectively. Adjusted odds ratios for both markers elevated versus neither elevated were 2.98 (95% confidence interval [CI], 2.04–4.36) in MIMIC-IV and 3.34 (95% CI, 0.97–11.54) in eICU. The glucose association in MIMIC-IV was nonlinear (*p* = 0.005), whereas the WBC curve varied with the spline specification. Among 229 eICU patients with both markers measured, adding the joint category to the clinical baseline model changed the area under the receiver operating characteristic curve from 0.796 to 0.811 (difference, 0.014; 95% CI, −0.012 to 0.039) and the Brier score, a measure of prediction error, by −0.0017 (95% CI, −0.0075 to 0.0041).

**Conclusion:**

Joint WBC–glucose elevation was associated with subsequent in-hospital mortality in MIMIC-IV, but estimates in the eICU cohort were imprecise. Additional predictive value remained uncertain, and continuous analyses did not establish a stable U-shaped association.

## 1 Introduction

Intracerebral hemorrhage (ICH) causes substantial mortality and disability. Neurological examination and imaging findings, including hematoma volume, location, and intraventricular extension, are central to prognostic assessment. Routine laboratory measurements cannot replace these disease-specific measures of severity, but they may provide additional information about the systemic response to acute injury (1–4).

An elevated white blood cell (WBC) count may reflect inflammatory activation, stress-related leukocyte mobilization, infection, or more extensive tissue injury. Hyperglycemia may reflect preexisting diabetes, neuroendocrine stress, treatment effects, or acute metabolic dysfunction. Previous studies have examined associations of inflammation, leukocyte responses, and blood glucose with outcomes after ICH (5–12). More recent studies have evaluated admission or serial WBC measurements and presenting or stress-related hyperglycemia. However, these studies differ in their populations, measurement windows, and outcomes (13–15).

Grouping patients based on whether one or both markers exceed specified thresholds offers a simple way to describe their joint status. However, values below these upper thresholds are not necessarily physiologically normal. Higher mortality among patients with both markers elevated also does not, by itself, demonstrate an interaction between the markers or show that they improve prognostic prediction. A separate question is whether mortality risk changes steadily across the range of each marker or follows a more complex pattern.

Our primary objective was to assess the association between joint early WBC–glucose elevation and subsequent in-hospital mortality among patients with ICH who were alive and hospitalized 24 h after intensive care unit (ICU) admission. This time point served as the landmark for assessment. Secondary objectives were to characterize the shape of each marker’s association with mortality and to determine whether the markers improved prediction beyond neurological status and a common clinical baseline model. Analyses added during peer review, including simpler models based on the Glasgow Coma Scale (GCS) and reconstructed lymphocyte counts, are identified separately. The study was not intended to establish admission treatment thresholds or an ICH severity score that could replace imaging.

## 2 Methods

### 2.1 Data sources and cohort definition

This retrospective study used harmonized patient-level extracts from the Medical Information Mart for Intensive Care IV (MIMIC-IV), specified as version 3.1, and the eICU Collaborative Research Database (eICU-CRD), version 2.0 (16, 17). MIMIC-IV was the primary cohort for association analyses and prediction-model development. The eICU database provided an exploratory assessment across multiple centers. Because eICU had already been used in earlier exploratory analyses, it was not considered an untouched dataset for confirmatory external validation. Reporting was guided by the Strengthening the Reporting of Observational Studies in Epidemiology (STROBE) statement and updated guidance for reporting regression and machine learning prediction models (18, 19).

After the initial selection of adult patients and eligible admissions, the extracts contained 1,644 MIMIC-IV and 2,208 eICU records. ICH was defined by the International Classification of Diseases, Ninth Revision (ICD-9) code 431 or Tenth Revision (ICD-10) code I61.x after code formatting was standardized. This definition retained 1,637 MIMIC-IV and 264 eICU records. Records identified only by broader unspecified intracranial hemorrhage codes or diagnosis text were excluded from the primary eICU cohort. The initial eICU extraction had selected one hemorrhage-related ICU admission per patient and required an ICU stay longer than one day. Supplementary Table S1 describes cohort selection and the limitations of the available extracts.

### 2.2 Landmark, outcome, and measurement window

Prediction began 24 h after ICU admission. In MIMIC-IV, the hospital discharge time and any recorded death time had to occur after this landmark. Thirty-one records did not meet these criteria. We did not use the ICU discharge timestamp because it conflicted with hospital discharge or death times in some records. For the eICU cohort, we retained the documented requirement of an ICU stay longer than 1 day; rounded length-of-stay values were not interpreted as exact timestamps. The outcome was in-hospital death after the landmark, excluding deaths during the first ICU day. The estimates therefore apply to patients who were alive and still hospitalized at the landmark, not to all patients presenting with ICH.

WBC, laboratory glucose, blood pressure, and other laboratory variables were the first values recorded during ICU hours 0–24. GCS was the lowest score recorded during the same window and could therefore reflect deterioration, sedation, intubation, or treatment during the first day. The extracts did not retain individual laboratory timestamps, the first GCS score, or all extraction mappings. We could not independently confirm whether measurements were obtained simultaneously or before treatment. Hospital identifiers were also unavailable in the eICU database. For a *post hoc* data-quality check, the MIMIC-IV source-event tables were reviewed for WBC measurements of the same order of magnitude as the three analytic values ≥100 × 10^9^/L. Repeated measurements in that range were recorded in K/μL. We did not re-extract or replace analytic variables or cohorts.

### 2.3 Exposure definitions and analytic samples

WBC elevation was defined as >10 × 10^9^/L and glucose elevation as >140 mg/dL. Patients with both measurements were assigned to four mutually exclusive groups: neither marker elevated, WBC elevation only, glucose elevation only, or both markers elevated. Values at or below the threshold were classified as not elevated. We used the term “neither marker elevated” rather than “normal” because this group could include patients with low WBC or glucose values. The secondary marker count was 0 when neither marker was elevated, 1 when either marker alone was elevated, and 2 when both were elevated. Patients missing either measurement were not classified as having zero elevated markers.

The primary association analyses included patients with complete data for both markers and the relevant cohort-specific adjustment variables. Prediction comparisons instead included all patients with both markers measured; missing baseline predictors were imputed using values learned from the training data. These different approaches explain why the association and prediction samples differ. Blood pressure values at or below zero were treated as missing, including one eICU systolic blood pressure (SBP) value of zero. High WBC values were retained in the primary analyses, even when influential. The association adjustment variables and the prediction baseline variables are specified separately below.

### 2.4 Primary association and GCS-based regression analyses

The four-group exposure was entered as a single categorical variable, with neither marker elevated as the reference category. Each cohort was analyzed in one multivariable model containing all four groups; we did not fit separate regressions to pairs of groups. The MIMIC-IV model used maximum likelihood logistic regression adjusted for age, sex, minimum GCS score, SBP, platelet count, creatinine, and recorded diabetes. The eICU model adjusted for age, sex, minimum GCS, and SBP. Because the eICU complete-case sample contained only 37 deaths, we used Firth bias-reduced logistic regression to reduce small-sample estimation bias. This method does not compensate for limited information (20, 21).

We report odds ratios (ORs), Wald 95% confidence intervals (CIs), two-sided *p* values, and coefficients for all adjustment variables. Wald intervals and tests were retained for consistency with the submitted table. Profile-penalized intervals and tests, which provide an alternative assessment of uncertainty in the small eICU sample, are also reported for all three category comparisons. Overall tests assessed whether the four-group classification was associated with mortality, rather than testing only one group against the reference category. We used likelihood ratio tests in MIMIC-IV and penalized likelihood ratio tests in the eICU cohort. The latter retained the full-design Jeffreys penalty when imposing coefficient restrictions, as detailed in the Supplementary materials. These models estimate adjusted prognostic associations, not causal effects.

In response to peer review, we reported the distribution of minimum GCS scores and its univariable association with mortality among all patients with GCS data. We then fit simpler models to the same complete-case patients within each cohort: GCS score alone; GCS score with continuous WBC count and glucose level; GCS score with two indicators of marker elevation; GCS score with the four-group classification; and GCS score with a three-group count of elevated markers. We also fit the continuous, binary, and count models using the original clinical adjustment variables.

The three-group count model compared one and two elevated markers to none, allowing each comparison to have a different OR. An additional exploratory ordinal model assumed the same change in log-odds from zero to one and from one to two elevated markers. For models that assumed linear associations on the log-odds scale, ORs were reported per 5 × 10^9^/L increase in WBC count, per 50 mg/dL increase in glucose, and per 1-point decrease in GCS.

We calculated Wilson CIs for observed mortality proportions. Within each cohort, one overall Pearson chi-squared test compared survival status across the four exposure groups, and another compared the three marker-count groups. Because one expected cell count was below five in the eICU four-group table, we also performed a conditional Monte Carlo check using 100,000 simulated tables with the same row and column totals. These comparisons are unadjusted and do not establish independent associations. The additional regression analyses addressed reviewer requests and were not selected according to statistical significance. Their *p* values are exploratory and were not adjusted for multiple testing.

### 2.5 Nonlinear associations and reconstructed lymphocyte counts

To allow the associations to be non-monotonic rather than constrained to a straight line on the log-odds scale, WBC count and glucose level were modeled simultaneously using restricted cubic splines (RCS). Each spline had three knots, or points at which the curve could change curvature, at the cohort-specific 10th, 50th, and 90th percentiles. Each marker contributed one linear and one nonlinear term (22). We retained the samples, estimation methods, and clinical adjustment variables used in the primary association models. Overall tests assessed all terms for a marker together. Nonlinearity tests assessed each marker’s nonlinear term while retaining its linear term and the other marker’s spline.

The curves show adjusted ORs relative to a WBC count of 10 × 10^9^/L or glucose level of 140 mg/dL, with approximate pointwise Wald CIs. These values were reference points, not estimated minimum-risk values. The main MIMIC-IV figures display the original-sample 1st–99th percentile intervals for readability, although all observations were included in model fitting; full-range curves are provided in the Supplementary material. The eICU curves are restricted to observed values.

We specified two MIMIC-IV sensitivity analyses for this revision. One used a more flexible spline with four knots at the 5th, 35th, 65th, and 95th percentiles and retained all patients. The other retained the original three-knot locations after restricting both WBC counts and glucose levels to their original primary-sample 1st–99th percentile intervals. This central 98% analysis excluded records outside either interval and examined the influence of both tails; it did not redefine the primary cohort or imply that excluded values were erroneous. The primary analysis retained all observations. We report all specified curves and tests. A nonlinear curve need not change direction, and the pointwise CIs do not include uncertainty arising from the choice of spline specification.

We calculated the absolute lymphocyte count (ALC) by multiplying WBC count (×10^9^/L) by the lymphocyte percentage and dividing by 100. The ALC remained missing if either component was missing or the percentage was outside the range of 0–100. We did not use the stored absolute count fields because their scaling was inconsistent. Lymphopenia was defined as reconstructed ALC < 1.0 × 10^9^/L, using the threshold reported by Morotti and colleagues (23). The extracts did not establish whether the WBC count and lymphocyte percentage came from the same blood sample.

The MIMIC-IV lymphopenia analysis retained its original clinical adjustment variables. Because the eICU subset had only 16 deaths, its primary Firth model adjusted for age and GCS score; the original four-variable adjustment set was used in a sensitivity analysis. Each unadjusted analysis used the same patients as its corresponding adjusted analysis. These models did not additionally adjust for total WBC count or glucose level. They therefore do not determine whether the WBC count association is independent of lymphocyte composition.

### 2.6 Secondary prediction models and performance evaluation

To determine whether WBC count and glucose level improved prediction, we used two baseline models: minimum GCS score alone and a common clinical model containing age, sex, minimum GCS score, SBP, diastolic blood pressure, platelets, hemoglobin, creatinine, sodium, and potassium. Each baseline model was extended by adding either both natural-log-transformed markers, both RCS-modeled markers, or the four-group joint classification. The clinical model was also extended with log WBC count alone or log glucose level alone. The two principal comparisons evaluated each baseline model against the same model with the joint classification added. All other comparisons were exploratory. These prediction models were separate from the regressions used to estimate adjusted associations.

We used L2-penalized, or ridge, logistic regression to reduce overfitting by shrinking coefficients toward zero. Data preparation was performed separately within each training subset. Missing baseline measurements were replaced with training-subset medians, and binary missingness indicators were added only for baseline variables with missing values in that training subset. Continuous features, including log-transformed values and spline terms, were centered and scaled. Sex and binary indicators were not standardized. The joint classification was encoded using four unscaled indicators, one for each group. Prediction model spline knots were calculated within the training subset at the 10th, 50th, and 90th percentiles.

The parameter C controls the inverse strength of the ridge penalty. We selected C from 0.03, 0.1, 0.3, 1, and 3 to maximize average precision. Internal evaluation used nested cross-validation, which separates parameter selection from performance assessment. In each of the three repetitions, the data were divided into five outer folds. Each outer fold was held out for evaluation while the remaining folds were used for training. Within that training sample, three-fold inner cross-validation selected C. All models used the same splits. Performance for each repetition was calculated using the held-out predictions for all patients. Standard deviations (SDs) across repetitions describe variation between splits, not CIs.

For evaluation in the eICU cohort, we selected C using five-fold cross-validation in the complete MIMIC-IV development sample. The final model was then fitted to that entire sample and applied to the eICU cohort without retraining or recalibration.

We evaluated discrimination, prediction error, and calibration. The area under the receiver operating characteristic curve (AUROC) measures how well a model distinguishes between patients who die and those who survive. Average precision (AP) summarizes the precision of predicted deaths across levels of sensitivity; it is not the trapezoidal area under the precision–recall curve. The Brier score is the average squared difference between each predicted probability and the observed outcome, with lower values indicating less error. Calibration refers to the agreement between predicted probabilities and observed mortality (24–26).

Calibration in the large assessed systematic overprediction or underprediction while fixing the prediction-logit slope at one. In a separate regression, an intercept and slope were estimated together to assess calibration across the risk range. These analyses evaluated calibration but did not change the predictions. Scaled Brier scores, complete calibration results, model settings, and individual-fold results are provided in the Supplementary material and aggregate analysis files.

We estimated external 95% percentile CIs and paired differences between models using the same 2,000 patient-level bootstrap samples, with predictions held fixed. Within each resample, performance was calculated for both models, and the difference was recorded. The intervals therefore describe uncertainty in the eICU evaluation conditional on the fit models; they do not include uncertainty from repeating model development or account for clustering by hospital, because hospital identifiers were unavailable. An interval spanning zero was not interpreted as evidence of equivalence or clinical usefulness (27).

Sensitivity analyses examined the original threshold combinations (WBC counts of 10, 11, or 12 × 10^9^/L with glucose levels of 140 or 180 mg/dL), added anion gap back to the clinical baseline model, and included all landmark patients. In the all-patient analysis, missing marker values were imputed using training data, and both compared models received the same separate indicators for missing WBC counts and glucose levels.

During revision, the prediction analyses were rebuilt with explicit definitions of preprocessing, coding, splines, and validation. A fully specified RCS replaced the incompletely documented B-spline used previously. The revised prediction results replace the earlier values throughout the manuscript, and Supplementary Table S11 compares the two sets of results. We do not claim exact reproduction of the historical prediction results. Random-forest and Shapley additive explanations (SHAP) analyses were removed to focus the manuscript on adjusted associations, association shapes, and additional predictive value; model-attribution results are no longer used as evidence.

### 2.7 Statistical interaction and software

We examined statistical interaction by expressing the four-group association model in terms of two binary elevation indicators and their product. The interaction OR measures whether the joint association departs from the product of the two individual ORs. It does not measure interaction on the additive-risk scale or establish a biological mechanism. We used ordinary likelihood-ratio tests in MIMIC-IV and full-design penalized likelihood-ratio tests in the eICU cohort, with eICU profile results provided separately. This is a different expression of the same four-group model, not an independent validation.

Revision analyses used Python 3.13.5, NumPy 2.3.5, SciPy 1.17.0, scikit-learn 1.8.0, and statsmodels 0.14.6, together with a numerically checked Firth implementation in NumPy/SciPy. The reproducibility material in the Supplementary material includes code, aggregate results, model parameters, and software records. It contains no patient-level data or individual predictions.

## 3 Results

### 3.1 Cohorts and neurological status

The 24-h landmark cohorts included 1,606 patients in MIMIC-IV with 332 subsequent in-hospital deaths and 264 eICU patients with 42 deaths. Both markers were measured in 1,590 patients in MIMIC-IV with 325 deaths and 229 eICU patients with 40 deaths (Figure 1). After excluding patients with missing or unusable adjustment variables, the primary association samples comprised 1,589 patients with 324 deaths and 221 patients with 37 deaths, respectively. Table 1 describes the landmark cohorts; Supplementary Table S1 reports selection and missingness. Anion gap had the largest reported difference in distribution between databases, with an absolute standardized mean difference of 1.36.

**Figure 1:**
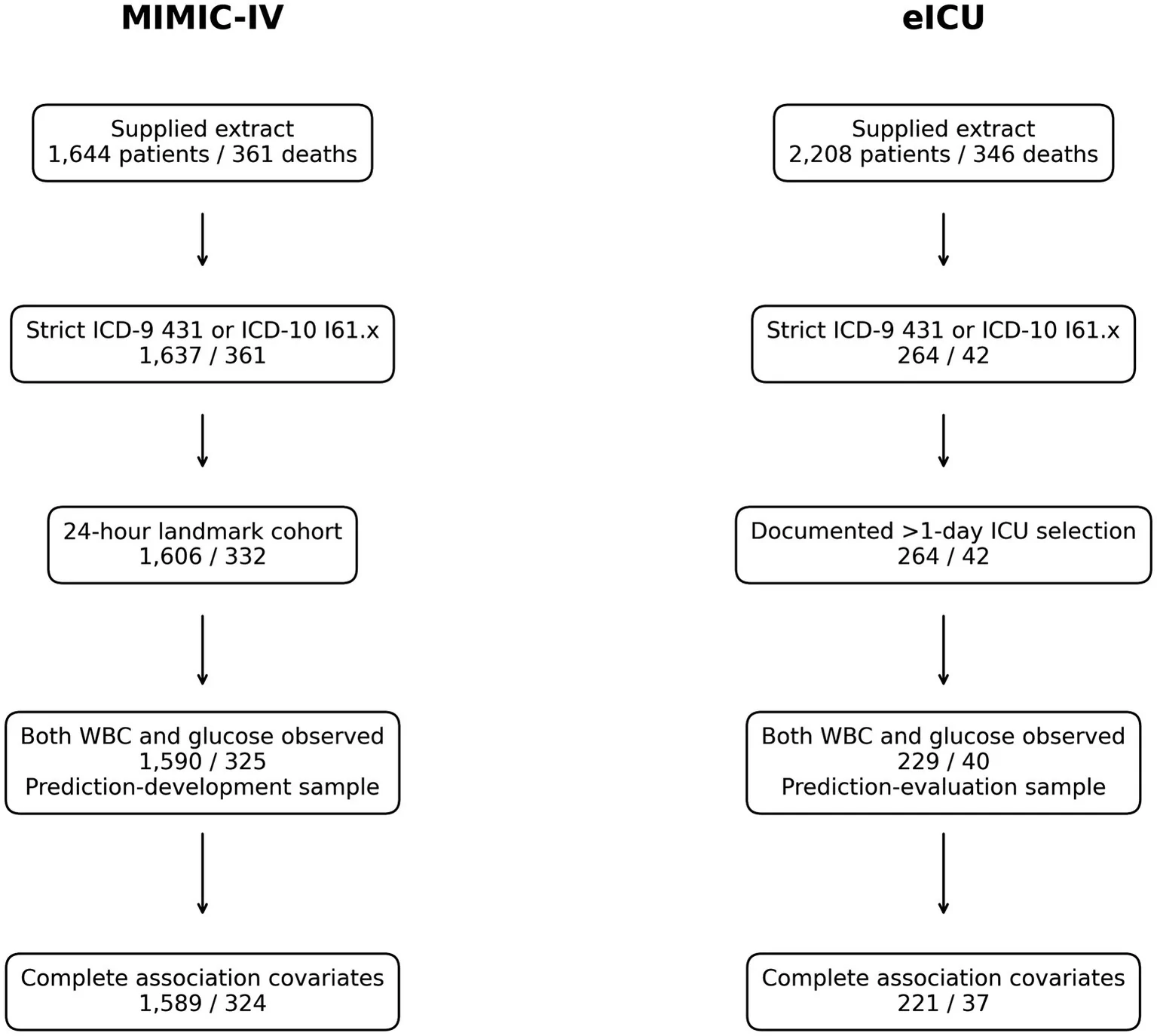
Cohort selection and analysis samples. Counts are patients/deaths. The MIMIC-IV landmark criteria excluded 31 records: 24 with recorded death within 24 h and seven other early-discharge records. Five of the latter had an eventual hospital-death flag and were not classified as surviving discharges. eICU retained the documented ICU-stay restriction from the original extraction; exact laboratory timestamps were unavailable. Prediction analyses imputed missing baseline covariates, whereas association analyses required complete, usable adjustment variables. ICD, International Classification of Diseases; ICU, intensive care unit; WBC, white blood cell count.

**Table 1:** Characteristics of the 24-h landmark cohorts.

Characteristic	MIMIC-IV (*n* = 1,606)	eICU (*n* = 264)	SMD
Age, years	68.9 (57.6–80.2)	64.0 (54.0–77.0)	0.18
Minimum GCS	12.0 (8.0–14.0)	13.0 (7.0–15.0)	0.09
First WBC, ×10^9^/L	10.2 (7.7–13.6)	10.2 (7.9–12.7)	0.11
First glucose, mg/dL	134.0 (111.0–165.0)	125.0 (108.0–155.8)	0.08
First systolic BP, mmHg	138.0 (122.0–152.0)	148.0 (136.0–163.2)	0.52
First diastolic BP, mmHg	74.0 (63.0–85.0)	75.5 (66.0–89.2)	0.17
Platelets, ×10^9^/L	209.0 (162.5–269.0)	205.0 (173.0–255.8)	0.09
Hemoglobin, g/dL	12.6 (11.0–14.0)	12.9 (11.5–14.4)	0.18
Creatinine, mg/dL	0.9 (0.7–1.1)	0.9 (0.7–1.3)	0.16
Anion gap, mEq/L	15.0 (13.0–17.0)	10.0 (8.0–13.0)	1.36
Sodium, mmol/L	139.0 (137.0–141.0)	140.0 (138.0–142.0)	0.20
Potassium, mmol/L	4.0 (3.7–4.4)	3.7 (3.4–4.0)	0.52
Male sex	873 (54.4%)	144 (54.5%)	0.00
Subsequent in-hospital death	332 (20.7%)	42 (15.9%)	0.12

Values are medians (interquartile ranges) or *n* (%). GCS, Glasgow Coma Scale; WBC, white blood cell count; BP, blood pressure; SMD, absolute standardized mean difference. These descriptive estimates are unchanged from the submitted table because the landmark cohorts are unchanged. Anion gap is excluded from the primary prediction baseline and included only in sensitivity analysis. The samples used for individual models differ from the full cohorts shown in Table 1.

GCS was available for 1,605 patients in MIMIC-IV, including 331 deaths, and 258 eICU patients, including 40 deaths. In MIMIC-IV, median GCS score was 13 (interquartile range [IQR], 9–14) among survivors and 7 (IQR, 3.5–13) among patients who died. The corresponding values in eICU were 14 (IQR, 10–15) and 4 (IQR, 3–7). The univariable OR per one-point decrease in GCS score was 1.23 (95% CI, 1.19–1.27) in MIMIC-IV and 1.34 (95% CI, 1.23–1.47) in eICU (both *p* < 0.001; Supplementary Table S2).

### 3.2 Joint elevation and full-cohort adjusted associations

Mortality in the neither-elevated, WBC-only, glucose-only, and both-elevated groups was 12.3%, 17.3%, 23.3%, and 31.6% in MIMIC-IV and 8.4%, 19.2%, 19.2%, and 29.8% in the eICU cohort, respectively (Table 2). Overall Pearson chi-square *p* values for the four-group classifications were <0.001 and 0.020. The eICU Monte Carlo check gave a similar result (*p* = 0.01952). Across zero, one, and two elevated markers, mortality was 12.3%, 19.7%, and 31.6% in MIMIC-IV and 8.4%, 19.2%, and 29.8% in the eICU cohort. These are unadjusted comparisons.

**Table 2:** Observed mortality by joint elevation and number of elevated markers.

Cohort	Scheme	Category	*N*	Deaths	Mortality % (95% CI)	Overall *p*
MIMIC-IV	Four categories	Neither marker elevated	504	62	12.3 (9.7–15.5)	<0.001
		WBC elevation only	405	70	17.3 (13.9–21.3)	
		Glucose elevation only	270	63	23.3 (18.7–28.7)	
		Both markers elevated	411	130	31.6 (27.3–36.3)	
MIMIC-IV	Three count categories	0 elevated markers	504	62	12.3 (9.7–15.5)	<0.001
		1 elevated marker	675	133	19.7 (16.9–22.9)	
		2 elevated markers	411	130	31.6 (27.3–36.3)	
eICU	Four categories	Neither marker elevated	83	7	8.4 (4.1–16.4)	0.020
		WBC elevation only	73	14	19.2 (11.8–29.7)	
		Glucose elevation only	26	5	19.2 (8.5–37.9)	
		Both markers elevated	47	14	29.8 (18.7–44.0)	
eICU	Three count categories	0 elevated markers	83	7	8.4 (4.1–16.4)	0.007
		1 elevated marker	99	19	19.2 (12.6–28.0)	
		2 elevated markers	47	14	29.8 (18.7–44.0)	

WBC, white blood cell count; CI, confidence interval. WBC elevation is >10 × 10^9^/L; glucose elevation is >140 mg/dL. The one-elevated-marker group combines WBC elevation only and glucose elevation only. “Neither elevated” does not imply normal physiological values. Patients missing either marker are excluded from this table. CIs are Wilson intervals. Each *p* value is an overall Pearson chi-square test for the complete cohort/classification block, not a test for the reference group alone. All comparisons are unadjusted.

Relative to neither marker elevated, adjusted ORs in MIMIC-IV were 1.67 (95% CI, 1.12–2.49; *p* = 0.012) for WBC elevation only, 1.78 (1.15–2.75; *p* = 0.009) for glucose elevation only, and 2.98 (2.04–4.36; *p* < 0.001) for both markers elevated. The corresponding eICU Firth estimates were 1.95 (0.58–6.58; *p* = 0.282), 4.64 (1.02–21.03; *p* = 0.047), and 3.34 (0.97–11.54; *p* = 0.057). Tables 3 and 4 present coefficients for every category and clinical adjustment variable. The overall classification test was significant in MIMIC-IV (3 degrees of freedom; *p* < 0.001) but not in the eICU cohort (penalized likelihood-ratio *p* = 0.141). Thus, the nominally significant glucose-only comparison should not be interpreted as confirmation of the overall classification in eICU. Profile-penalized results for all three eICU comparisons are reported in Supplementary Table S4.

**Table 3:** Full-cohort adjusted association model — MIMIC-IV.

Exposure/adjustment variable	Group N/deaths	Adjusted OR (95% CI)	Wald *p*
Neither marker elevated	504/62	Reference	—
WBC elevation only	405/70	1.67 (1.12–2.49)	0.012
Glucose elevation only	269/62	1.78 (1.15–2.75)	0.009
Both markers elevated	411/130	2.98 (2.04–4.36)	<0.001
Age, per 10-year increase	—	1.27 (1.16–1.40)	<0.001
Male vs. female	—	0.90 (0.68–1.18)	0.434
Minimum GCS, per 1-point decrease	—	1.21 (1.17–1.26)	<0.001
First SBP, per 10-mmHg increase	—	0.97 (0.92–1.03)	0.286
Platelets, per 100 × 10^9^/L increase	—	0.73 (0.62–0.86)	<0.001
Creatinine, per 1-mg/dL increase	—	1.28 (1.13–1.44)	<0.001
Recorded diabetes vs. none	—	1.06 (0.77–1.46)	0.720

Model-level sample: 1,589 patients; 324 subsequent in-hospital deaths. All four exposure categories were included in this single model. Overall exposure test (3 degrees of freedom): likelihood ratio, statistic 33.6421; *p* < 0.001.

OR, odds ratio; CI, confidence interval; GCS, Glasgow Coma Scale; SBP, systolic blood pressure; WBC, white blood cell count. All category and adjustment-variable coefficients were estimated simultaneously. Group counts refer to patients within this complete-case model, not to separate regression samples. A dash indicates that a group count is not applicable to an adjustment variable. The intercept and unrounded coefficients are available in the aggregate coefficient file.

The model used maximum-likelihood logistic regression. One patient in the glucose-elevation-only group had missing GCS, giving 269/62 patients/deaths in this table compared with 270/63 in Table 2. Counts for the other groups are unchanged.

**Table 4:** Full-cohort adjusted association model — eICU.

Exposure/adjustment variable	Group N/deaths	Adjusted OR (95% CI)	Wald *p*
Neither marker elevated	81/7	Reference	—
WBC elevation only	69/11	1.95 (0.58–6.58)	0.282
Glucose elevation only	25/5	4.64 (1.02–21.03)	0.047
Both markers elevated	46/14	3.34 (0.97–11.54)	0.057
Age, per 10-year increase	—	1.20 (0.88–1.64)	0.249
Male vs. female	—	3.23 (1.30–8.01)	0.012
Minimum GCS, per 1-point decrease	—	1.32 (1.19–1.45)	<0.001
First SBP, per 10-mmHg increase	—	0.81 (0.69–0.96)	0.014

Model-level sample: 221 patients; 37 subsequent in-hospital deaths. All four exposure categories were included in this single model. Overall exposure test (3 degrees of freedom): full-design penalized likelihood ratio, statistic 5.4591; *p* = 0.141.

OR, odds ratio; CI, confidence interval; GCS, Glasgow Coma Scale; SBP, systolic blood pressure; WBC, white blood cell count. All category and adjustment-variable coefficients were estimated simultaneously. Group counts refer to patients within this complete-case model, not to separate regression samples. A dash indicates that a group count is not applicable to an adjustment variable. The intercept and unrounded coefficients are available in the aggregate coefficient file.

The model used Firth bias-reduced logistic regression. Wald standard errors were calculated from the inverse expected information matrix at the Firth estimate. The sample decreased from 229/40 patients/deaths with both markers measured to 221/37 after checking GCS and SBP, including treatment of one zero SBP value as missing. Supplementary Table S4 reports profile-penalized results for all three category comparisons. For both markers elevated, the lower profile CI limit was 0.9980 and *p* = 0.0504; this sensitivity analysis does not establish statistical significance.

### 3.3 GCS-based models and the number of elevated markers

In the common MIMIC-IV association sample, the model containing GCS, continuous WBC, and continuous glucose level gave a WBC count OR of 1.04 per 5 × 10^9^/L increase (95% CI, 0.99–1.09; *p* = 0.110) and a glucose level OR of 1.27 per 50 mg/dL increase (1.16–1.39; *p* < 0.001). Neither corresponding estimate was statistically significant in eICU: the WBC OR was 1.49 (0.88–2.54; *p* = 0.140), and the glucose level OR was 1.21 (0.86–1.71; *p* = 0.279). These estimates assume linear associations on the log-odds scale and do not rule out nonlinear associations.

After adjustment for the original clinical variables, the three-group count model gave ORs of 1.72 (95% CI, 1.20–2.44; *p* = 0.003) for one elevated marker and 2.98 (2.04–4.36; *p* < 0.001) for two elevated markers in MIMIC-IV, each relative to none. The corresponding eICU estimates were 2.35 (0.74–7.46; *p* = 0.149) and 3.23 (0.94–11.08; *p* = 0.062). Supplementary Table S3 reports all GCS-based, continuous, binary, and count-model results, including nonsignificant estimates. Overall tests are reported in Supplementary Table S4.

### 3.4 Continuous WBC and glucose associations

In the primary three-knot MIMIC-IV model, glucose level was associated with subsequent mortality (overall *p* < 0.001), and the association was nonlinear (*p* = 0.005). The fit curve rose with increasing glucose level but changed slope; it did not demonstrate a U-shaped relationship (Figure 2). At a glucose level of 200 versus 140 mg/dL, the adjusted OR was 1.40 (95% CI, 1.22–1.59). Nonlinearity remained evident in the four-knot and central 98% sensitivity analyses (*p* = 0.013 and *p* = 0.024, respectively). Only nine patients had glucose <70 mg/dL, including one who died, limiting inference at the low end of the distribution.

**Figure 2:**
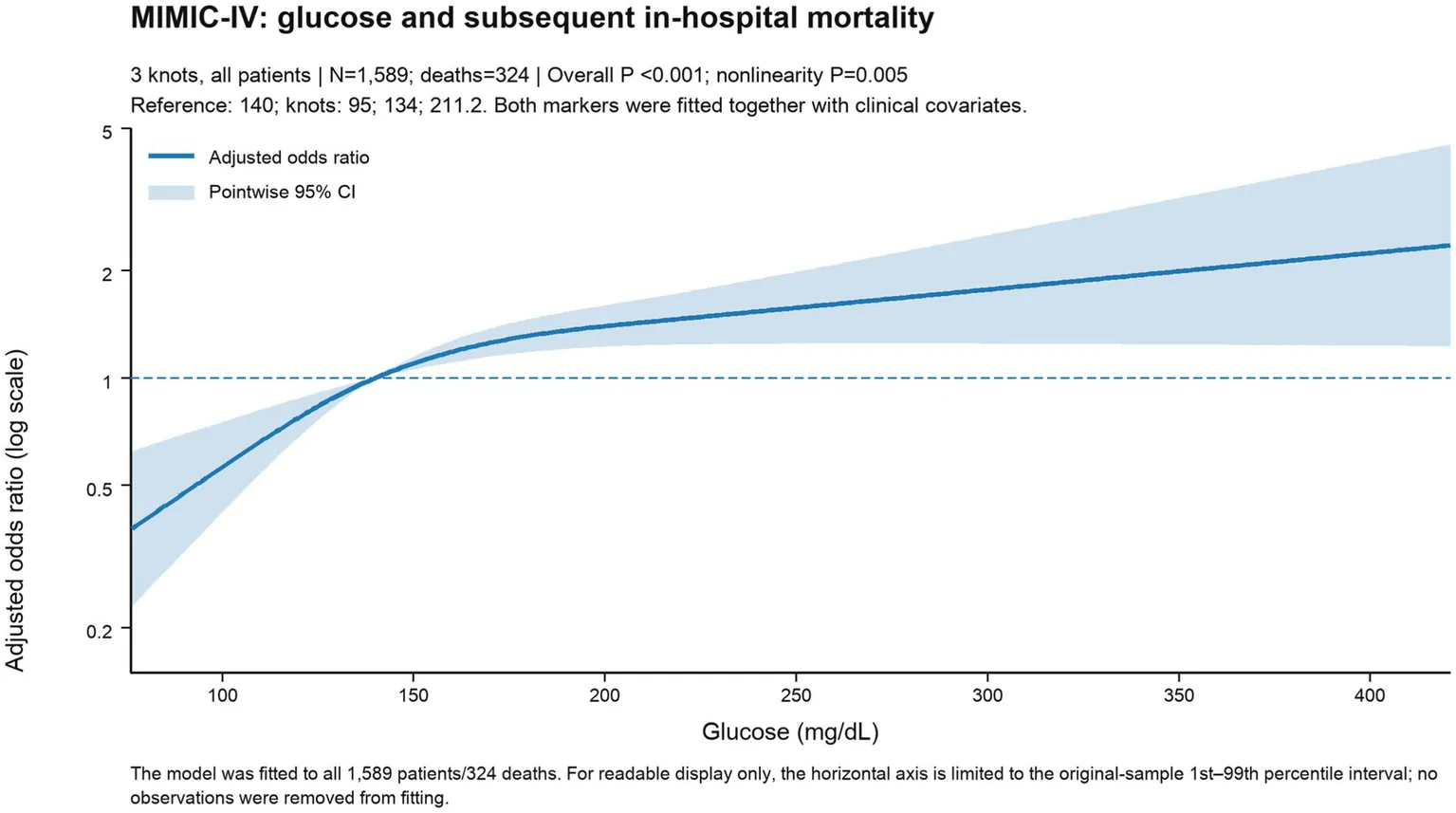
Adjusted association between glucose and mortality in MIMIC-IV. Glucose and WBC were modeled simultaneously using three-knot restricted cubic splines, with adjustment for age, sex, minimum GCS, SBP, platelets, creatinine, and diabetes (1,589 patients/324 deaths). ORs are relative to glucose 140 mg/dL; bands show approximate pointwise Wald 95% CIs. All observations were included in fitting; the horizontal display is limited to the original-sample 1st–99th percentile interval for readability. The reference value is not a treatment target. OR, odds ratio; CI, confidence interval; WBC, white blood cell count; GCS, Glasgow Coma Scale; SBP, systolic blood pressure. The model was fitted to all 1,589 patients/324 deaths. For readable display only, the horizontal axis is limited to the original-sample 1st–99th percentile interval; no observations were removed from fitting.

The primary MIMIC-IV WBC count model provided no clear evidence of an overall association (*p* = 0.131) or nonlinearity (*p* = 0.230; Figure 3). In contrast, the four-knot model using all patients showed nonlinearity (*p* < 0.001) and a fitted curve that changed direction. The central 98% analysis restricted both markers to a WBC count of 3.4–28.836 × 10^9^/L and glucose level of 76–420.84 mg/dL, excluding 61 patients, including 20 deaths. In that analysis, WBC was associated with mortality overall (*p* < 0.001), but its nonlinear term was not supported (*p* = 0.222); glucose nonlinearity persisted (*p* = 0.024). The primary analysis retained the WBC count maximum of 290.7 × 10^9^/L, and targeted review of MIMIC-IV source-event tables identified repeated WBC count measurements of the same order of magnitude in K/μL. Figure 4 compares all three specifications. The differences indicate that the WBC count curve depends on spline flexibility rather than supporting a single stable shape.

**Figure 3:**
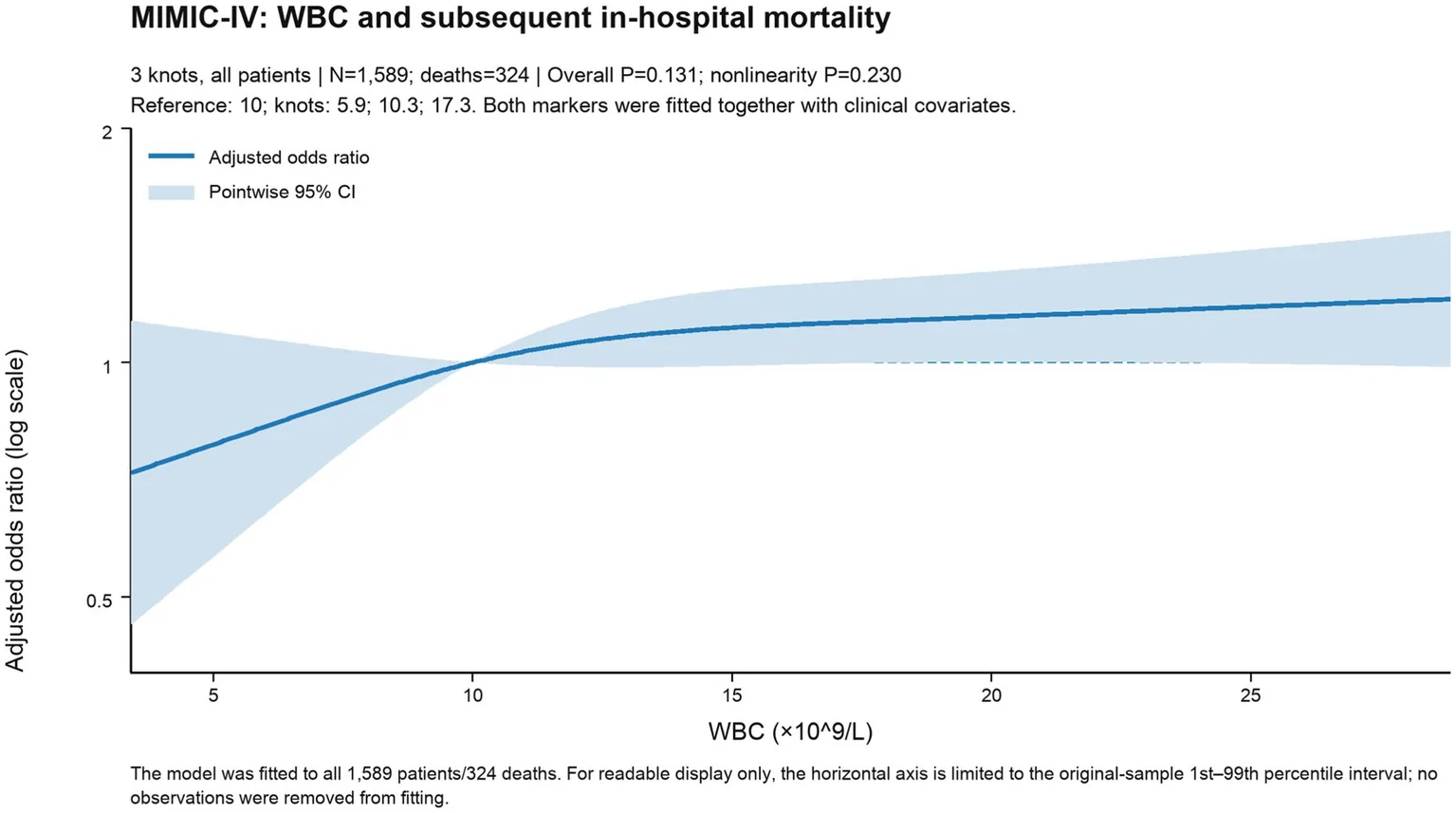
Adjusted association between WBC and mortality in MIMIC-IV. The primary model included three-knot splines for both WBC and glucose and the same seven clinical variables as Figure 2. ORs are relative to WBC 10 × 10^9^/L, with pointwise Wald 95% CIs. All WBC values were included in fitting; the horizontal display is limited to the original-sample 1st–99th percentile interval for readability. This curve should be considered alongside Figure 4 and the full-range and sensitivity curves in the supplement. WBC, white blood cell count; OR, odds ratio; CI, confidence interval. The model was fitted to all 1,589 patients/324 deaths. For readable display only, the horizontal axis is limited to the original-sample 1st–99th percentile interval; no observations were removed from fitting.

**Figure 4:**
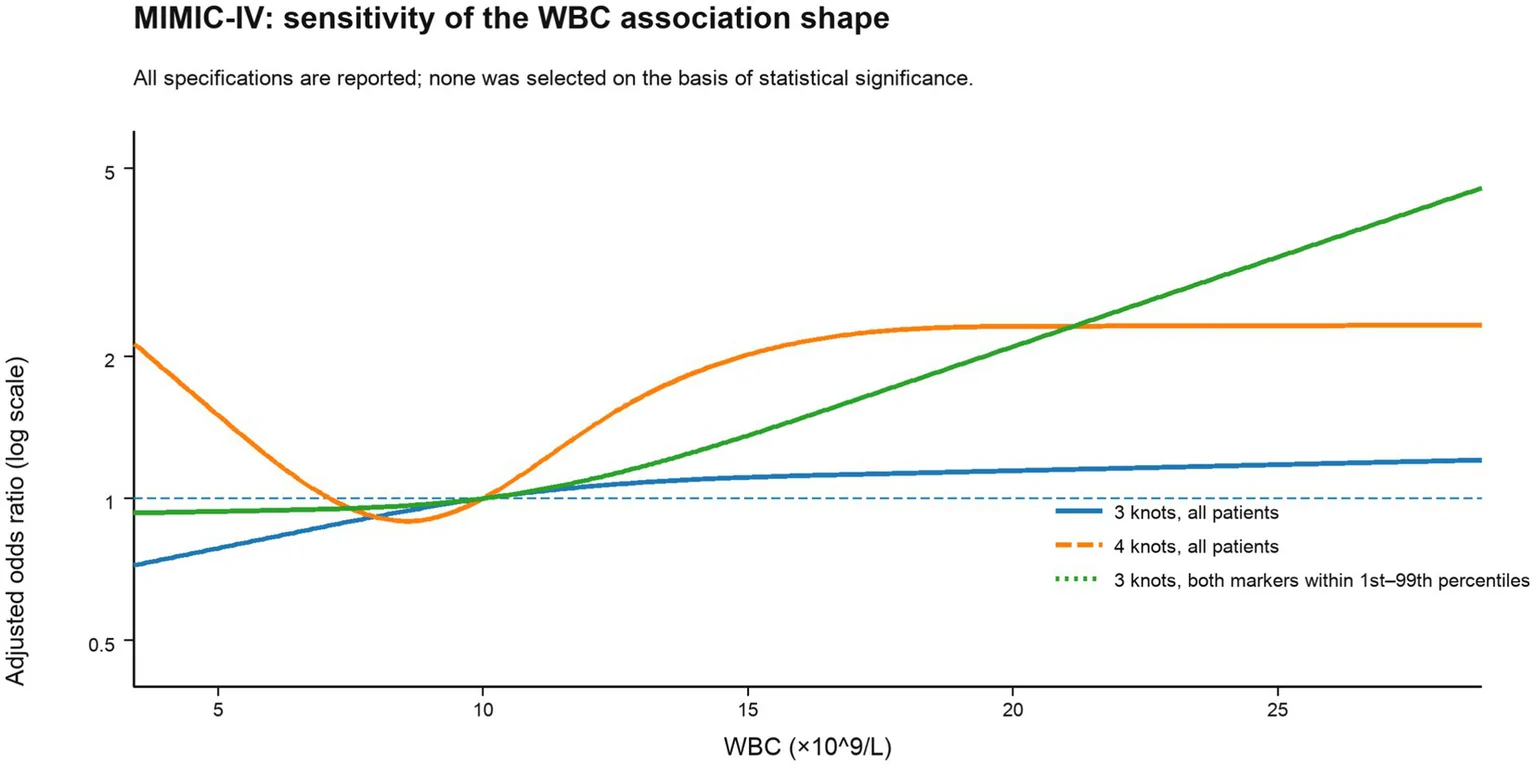
WBC association curves across the specified MIMIC-IV models. The curves compare the primary three-knot model, the four-knot model using all patients, and the three-knot model restricted to records in which both WBC and glucose were within their original-sample 1st–99th percentile intervals. These are sensitivity analyses, not independent cohorts or alternative primary results selected for significance. Only point estimates are shown; model-specific pointwise confidence bands are provided in Figure 3; Supplementary Figures S6, S8. WBC, white blood cell count; OR, odds ratio. Point estimates only. Separate figures and tables provide 95% CIs. The central 98% sensitivity excluded 61 patients/20 deaths; the primary model retained all observations. Reference values, knot locations and clinical adjustment were unchanged.

In the eICU cohort, *p* values for the overall association and nonlinearity were 0.071 and 0.064 for WBC count and 0.178 and 0.825 for glucose level. No eICU patient had a WBC count <4 × 10^9^/L, and only two had a glucose level <70 mg/dL; neither died. MIMIC-IV included 38 patients with WBC < 4 × 10^9^/L, including 10 deaths. Supplementary Table S5 reports all tests, knot locations, and low-range counts. Supplementary Figures S1–S9 show the eICU curves, full-range MIMIC-IV curves, and sensitivity analyses.

### 3.5 Reconstructed lymphopenia

The ALC could be reconstructed for 1,078/1,606 patients in MIMIC-IV (67.1%) and 125/264 eICU patients (47.3%). Of these patients, 437 (40.5%) and 47 (37.6%), respectively, had lymphopenia. Mortality with versus without lymphopenia was 25.2% (110/437) versus 17.5% (112/641) in MIMIC-IV and 14.9% (7/47) versus 12.8% (10/78) in the eICU cohort.

The adjusted MIMIC-IV analysis included 1,076 patients with 222 deaths and gave an OR of 1.54 (95% CI, 1.12–2.14; *p* = 0.009). The age- and GCS score-adjusted eICU model included 122 patients with 16 deaths and yielded an imprecise estimate (OR, 0.80; 95% CI, 0.24–2.70; *p* = 0.718). The eICU sensitivity analysis using the original four adjustment variables included 120 patients with 16 deaths and was also imprecise (OR, 1.33; 95% CI, 0.31–5.67; *p* = 0.702). Supplementary Table S6 reports availability, missingness, unadjusted results, and profile-penalized sensitivity results. A consistent lymphopenia association across both databases was not established.

### 3.6 Prediction beyond GCS and the clinical baseline

Across three repetitions of nested cross-validation in MIMIC-IV, mean AUROC from held-out predictions was 0.675 for the GCS score alone and 0.712 after adding the joint classification. For the full clinical baseline model, mean AUROC was 0.732 before and 0.750 after adding the classification. Mean Brier scores changed from 0.1421 to 0.1406 and from 0.1380 to 0.1363, respectively (Supplementary Table S7). These are internal assessments of the specified modeling procedure, not independent replications.

In the common eICU evaluation sample, the GCS baseline model and the model with the joint classification added had AUROCs of 0.803 and 0.811. The paired AUROC difference was 0.007 (95% CI, −0.024 to 0.041), and the Brier score difference was −0.0011 (95% CI, −0.0049 to 0.0027). For the clinical baseline model, AUROC changed from 0.796 to 0.811 after adding the classification; the AUROC and Brier-score differences were 0.014 (95% CI, −0.012 to 0.039) and −0.0017 (95% CI, −0.0075 to 0.0041), respectively. Neither principal comparison therefore established a clear improvement in the eICU cohort (Tables 5, 6; Figure 5). Differences were calculated before rounding and may not equal the subtraction of the displayed AUROCs.

**Table 5:** Exploratory eICU predictive performance.

Model	AUROC (95% CI)	AP (95% CI)	Brier (95% CI)
GCS baseline	0.803 (0.718 to 0.880)	0.471 (0.334 to 0.618)	0.1141 (0.0912 to 0.1392)
GCS + log pair	0.813 (0.733 to 0.880)	0.456 (0.330 to 0.630)	0.1158 (0.0926 to 0.1415)
GCS + RCS pair	0.821 (0.746 to 0.887)	0.462 (0.334 to 0.638)	0.1139 (0.0909 to 0.1392)
GCS + joint category	0.811 (0.726 to 0.884)	0.506 (0.360 to 0.666)	0.1130 (0.0899 to 0.1384)
Clinical baseline	0.796 (0.718 to 0.867)	0.473 (0.335 to 0.641)	0.1178 (0.0940 to 0.1440)
Clinical + log WBC	0.818 (0.749 to 0.879)	0.500 (0.362 to 0.665)	0.1154 (0.0924 to 0.1421)
Clinical + log glucose	0.807 (0.736 to 0.869)	0.455 (0.321 to 0.613)	0.1185 (0.0938 to 0.1457)
Clinical + log pair	0.818 (0.750 to 0.876)	0.458 (0.327 to 0.622)	0.1168 (0.0931 to 0.1432)
Clinical + RCS pair	0.810 (0.741 to 0.870)	0.458 (0.323 to 0.618)	0.1177 (0.0937 to 0.1446)
Clinical + joint category	0.811 (0.737 to 0.876)	0.470 (0.338 to 0.635)	0.1162 (0.0926 to 0.1430)

All rows evaluate the same 229 patients with 40 deaths, using models trained in MIMIC-IV and applied without updating. GCS, Glasgow Coma Scale; WBC, white blood cell count; RCS, restricted cubic spline; AUROC, area under the receiver operating characteristic curve; AP, average precision; CI, confidence interval. Log pair: natural-log-transformed WBC and glucose. Joint category: neither elevated, WBC elevation only, glucose elevation only, or both elevated. Thresholds are WBC >10 × 10^9^/L and glucose >140 mg/dL. The clinical baseline includes age, sex, minimum GCS, systolic and diastolic blood pressure, platelets, hemoglobin, creatinine, sodium, and potassium. Missingness indicators for numerical baseline variables are defined using training data. Patient-level bootstrap intervals are conditional on the fitted development models. Calibration results are in Supplementary Table S8.

**Table 6:** Paired eICU differences between prediction models.

Comparison	ΔAUROC (95% CI)	ΔAP (95% CI)	ΔBrier (95% CI)
GCS + log pair minus GCS baseline	0.010 (−0.027 to 0.051)	−0.016 (−0.087 to 0.098)	0.0017 (−0.0041 to 0.0077)
GCS + RCS minus GCS baseline	0.018 (−0.018 to 0.057)	−0.009 (−0.078 to 0.101)	−0.0002 (−0.0067 to 0.0069)
* GCS + category minus GCS baseline	0.007 (−0.024 to 0.041)	0.034 (−0.039 to 0.124)	−0.0011 (−0.0049 to 0.0027)
Clinical + log WBC minus Clinical baseline	0.021 (0.000 to 0.047)	0.027 (−0.033 to 0.086)	−0.0025 (−0.0064 to 0.0014)
Clinical + log glucose minus Clinical baseline	0.011 (−0.017 to 0.038)	−0.018 (−0.111 to 0.053)	0.0007 (−0.0052 to 0.0070)
Clinical + log pair minus Clinical baseline	0.021 (−0.011 to 0.054)	−0.016 (−0.111 to 0.061)	−0.0010 (−0.0073 to 0.0055)
Clinical + RCS minus Clinical baseline	0.013 (−0.014 to 0.040)	−0.015 (−0.099 to 0.054)	−0.0001 (−0.0054 to 0.0057)
* Clinical + category minus Clinical baseline	0.014 (−0.012 to 0.039)	−0.004 (−0.094 to 0.078)	−0.0017 (−0.0075 to 0.0041)
Clinical + category minus Clinical + log pair	−0.007 (−0.033 to 0.016)	0.012 (−0.052 to 0.082)	−0.0007 (−0.0052 to 0.0039)
Clinical + category minus Clinical + RCS	0.001 (−0.019 to 0.021)	0.011 (−0.053 to 0.080)	−0.0016 (−0.0059 to 0.0027)

Differences use unrounded values. Positive ΔAUROC/ΔAP and negative ΔBrier favor the first model. AUROC, area under the receiver operating characteristic curve; AP, average precision; CI, confidence interval; GCS, Glasgow Coma Scale; RCS, restricted cubic spline; WBC, white blood cell count. * Principal comparison of added predictive value. Every row uses the same 229 patients/40 deaths and the same 2,000 bootstrap samples. Intervals are calculated from model differences within each resample, not by subtracting separate CI limits. They are conditional on the fitted models and exclude uncertainty from model refitting and hospital clustering. Other comparisons are exploratory. The lower ΔAUROC limit for adding log WBC alone is exactly zero. Neither a nonsignificant difference nor an interval ending at zero establishes equivalence or clinical usefulness.

**Figure 5:**
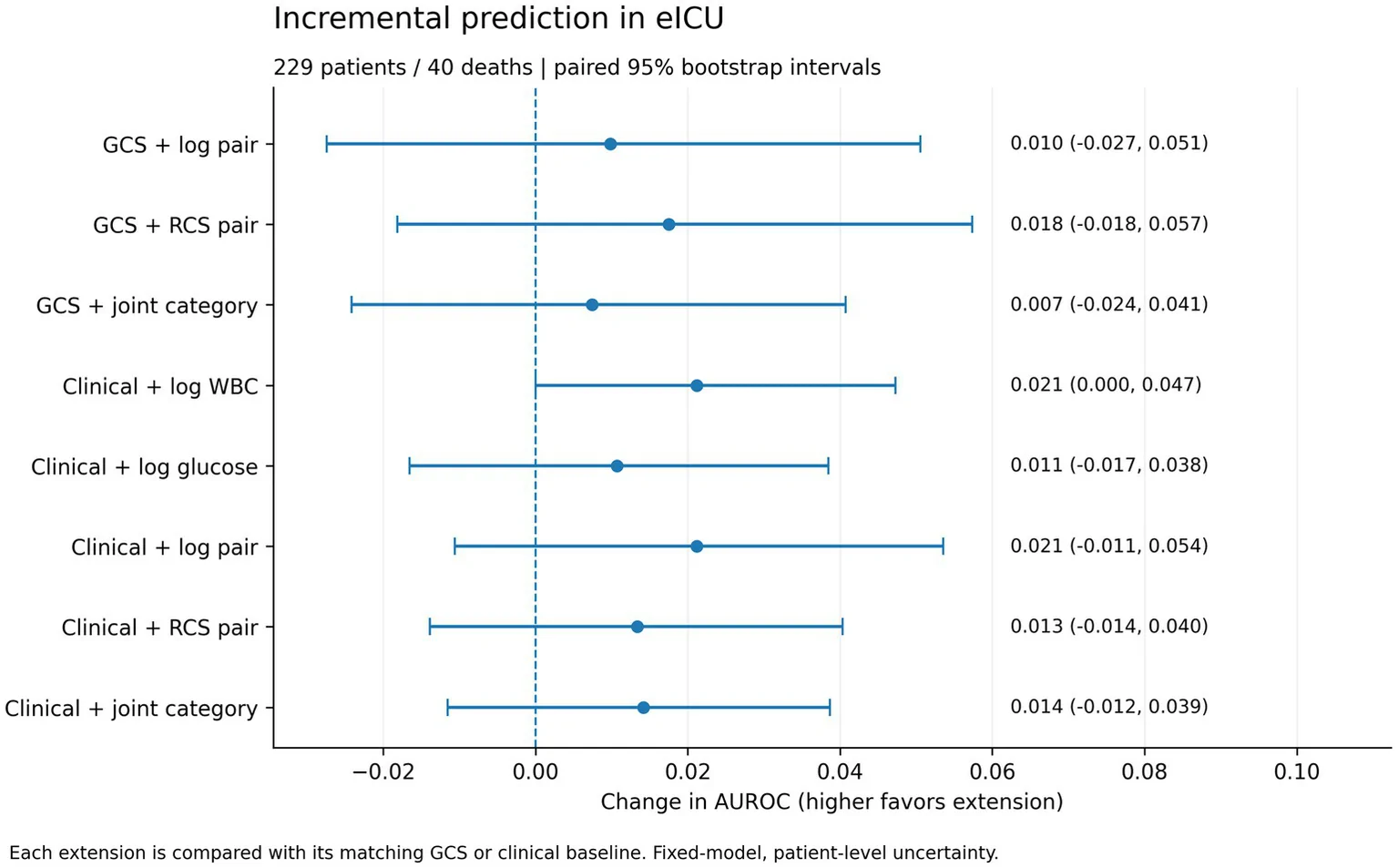
Changes in discrimination after adding WBC and glucose to the eICU prediction models. Each extended model is compared with its corresponding GCS or clinical baseline model in the same 229 patients with 40 deaths. Points show ΔAUROC; intervals are based on 2,000 paired patient-level bootstrap samples with MIMIC-IV-trained predictions held fixed. Both principal joint-category comparisons have intervals spanning zero. GCS, Glasgow Coma Scale; AUROC, area under the receiver operating characteristic curve; WBC, white blood cell count; RCS, restricted cubic spline. Each extension is compared with its matching GCS or clinical baseline. Fixed-model, patient-level uncertainty.

The clinical models containing both log-transformed markers or both RCS-modeled markers had eICU AUROCs of 0.818 and 0.810, respectively. Compared with these models, the joint classification model had AUROC differences of −0.007 (95% CI, −0.033 to 0.016) and 0.001 (95% CI, −0.019 to 0.021). These intervals do not establish equivalence. Adding log WBC alone to the clinical baseline model gave an exploratory ΔAUROC of 0.021 (95% CI, 0.000 to 0.047), with a lower interval endpoint of exactly zero. Its AP and Brier score difference intervals also included zero. Table 6 reports all comparisons.

Calibration estimates were imprecise. For the clinical baseline model and its joint classification extension, calibration-in-the-large estimates were −0.144 (95% CI, −0.522 to 0.195) and −0.108 (95% CI, −0.493 to 0.243), and slopes were 1.308 (95% CI, 0.904–1.868) and 1.149 (95% CI, 0.813–1.636), respectively. Figure 6 shows grouped calibration, and Supplementary Table S8 provides all diagnostics. Models were not recalibrated in eICU. In the sensitivity analysis including all landmark patients, the joint classification model had a lower AUROC than the log-pair model (difference, −0.030; 95% CI, −0.075 to 0.007; Supplementary Table S9). Alternative threshold and anion-gap comparisons were also imprecise.

**Figure 6:**
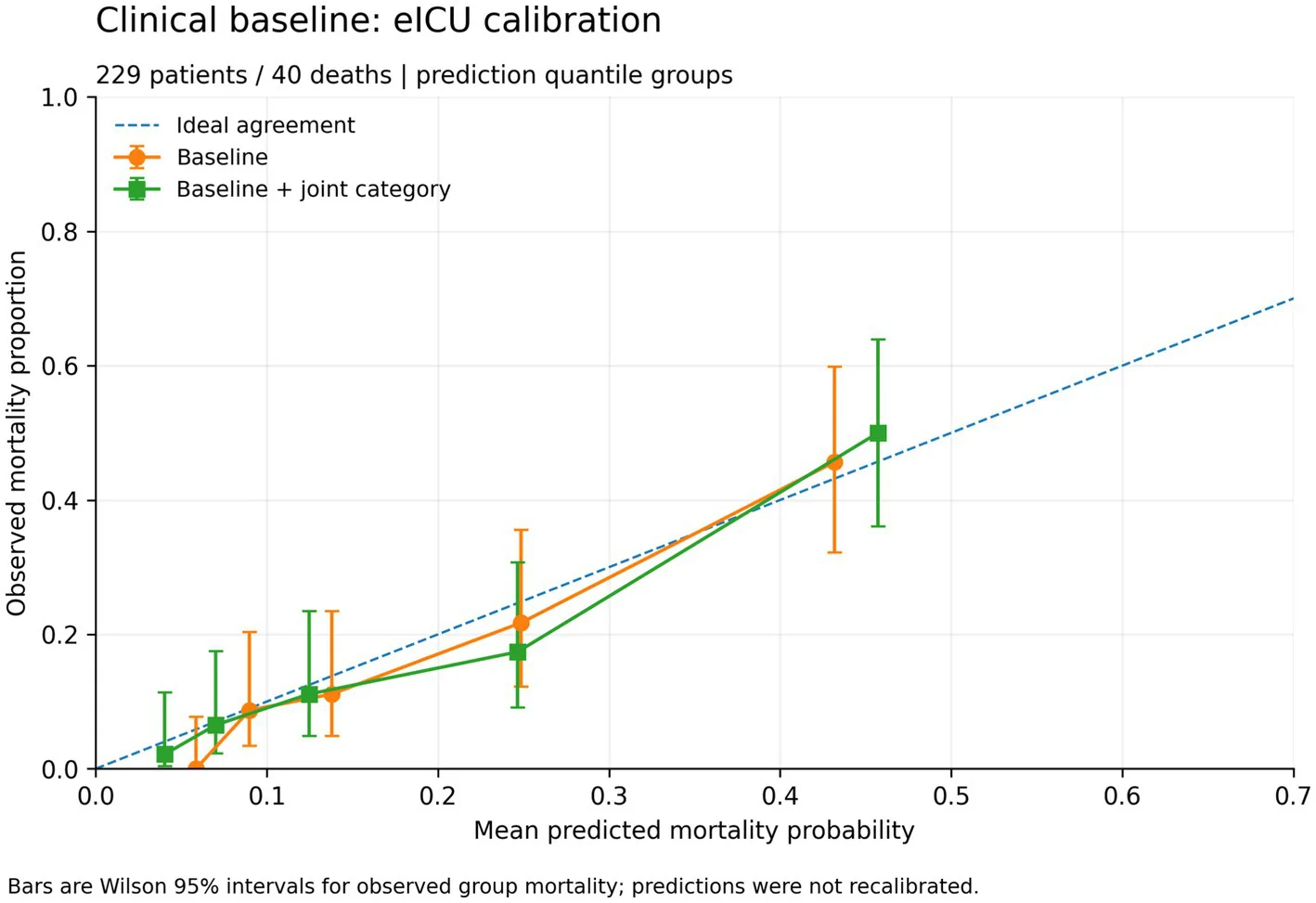
Calibration of the clinical baseline model and its joint-category extension in eICU. Patients are grouped by quantiles of predicted probability. Bars show Wilson 95% confidence intervals for observed mortality within each group, not simultaneous confidence bands for the entire calibration curve. Neither model was recalibrated. Calibration-in-the-large and slope estimates are reported in Supplementary Table S8. Bars are Wilson 95% intervals for observed group mortality; predictions were not recalibrated.

### 3.7 Statistical interaction

The product term analysis provided no clear evidence of interaction on the multiplicative OR scale. The interaction OR was 1.00 (95% CI, 0.58–1.74; likelihood ratio *p* = 0.989) in MIMIC-IV and 0.37 (0.06–2.28; penalized likelihood ratio *p* = 0.279) in the eICU cohort. The eICU profile-penalized estimate was similarly imprecise (Supplementary Table S10). These results do not establish the absence of biological synergy.

## 4 Discussion

This exploratory landmark study examined adjusted prognostic associations, the shapes of continuous associations, and additional predictive value as separate questions. Elevation of both markers was associated with higher subsequent mortality in the primary MIMIC-IV model. In the eICU cohort, estimates were imprecise, and the overall four-group test was not significant. The simpler regression models did not show uniformly significant associations for either marker. The nonlinear glucose level association in MIMIC-IV was more consistent across model specifications than the WBC count association. Neither cohort provided enough low-glucose level observations for a firm conclusion about risk in that range. Improvements in prediction beyond the GCS score or the clinical baseline model remained uncertain in the eICU cohort.

These findings are consistent with earlier interest in inflammatory and metabolic responses after ICH. Previous studies used admission, serial, or trial-based measurements and assessed different outcomes (11–14). Farenhorst et al. reported that the findings for presenting glucose depended on the outcome and adjustment variables, illustrating why glucose–outcome associations should not be regarded as interchangeable across studies (15). Our analysis adds a defined assessment point after the first ICU day and an interpretable joint classification. It does not establish a distinct biological phenotype or a classification sufficient for individual prognosis.

The reference group definition is important. Patients with low glucose levels or WBC counts can fall below both upper thresholds, so “neither elevated” does not mean physiologically normal. The American Heart Association/American Stroke Association guideline recommends attention to both high and low glucose levels after ICH, supporting examination of both ends of the distribution (1). Low-glucose observations were sparse in our cohorts. The broadly increasing fitted curves, together with their model-imposed behavior at the extremes, cannot establish that lower glucose levels are necessarily safer or identify a treatment target.

The WBC count findings were sensitive to model specification. A four-knot model produced a curve that changed direction, whereas the simpler three-knot model using all patients did not provide clear evidence of nonlinearity. The central 98% analysis also did not support WBC count nonlinearity. Reporting all specified models avoids selecting the most significant curve. The analyses support examining both low and high WBC count values but do not establish a stable U-shaped biological relationship. Belok et al. studied leukopenia in patients with suspected infection, and its association with mortality weakened after adjustment for organ dysfunction (28). Those findings do not establish sepsis as the explanation for low WBC in our ICH cohorts, particularly because infection data were not harmonized.

The reconstructed-ALC findings provide an exploratory assessment of leukocyte composition. Morotti et al. examined early lymphopenia, infectious complications, and 90-day outcomes after spontaneous ICH (23). Our ICU-based measurement window, subsequent in-hospital outcome, and reconstructed measure differ from that study. Only approximately two-thirds of patients in MIMIC-IV and one-half of eICU patients had the required measurements. We could not verify that the components came from the same blood sample or assume that missing data were random. Estimates from the small eICU subset were imprecise and did not consistently agree in direction. These analyses do not establish that lymphopenia mediates the WBC count association or that infection explains either marker’s association with mortality.

The number of elevated markers is a simplified description of joint status, not a causal dose. Combining the two groups with one elevated marker removes the distinction between WBC count elevation and glucose level elevation. Treating the count as an ordinal variable assumes equal changes in log-odds for each additional elevated marker. The four-group model therefore remains the primary clinical analysis, with categorical count and ordinal count results reported separately. The product-term analysis tests only interaction on the multiplicative odds scale; its imprecision and lack of statistical significance do not exclude biological interaction or interaction on other scales.

An adjusted association does not necessarily translate into useful improvement in prediction. Although the joint classification remained associated with mortality in MIMIC-IV, adding it to the GCS score or the clinical baseline model did not clearly improve discrimination, prediction error, or calibration in the eICU cohort (24, 25, 27). The average improvements seen during internal evaluation do not resolve this uncertainty. Nonsignificant comparisons also do not establish that categorization and continuous modeling are equivalent. In the analysis including all landmark patients, the joint-classification model had an unfavorable AUROC point estimate relative to the log-pair model. We did not evaluate a standalone tool based only on these biomarkers, thresholds for clinical decisions, or treatment benefit.

The landmark defines when prediction becomes available but restricts the population to which the results apply. Patients who died or left the hospital before the 24-h time point were excluded from subsequent risk estimates. Minimum first-day GCS score may reflect treatment and clinical deterioration as well as pretreatment neurological severity. Some excluded MIMIC-IV records had conflicting discharge and death information, and the original events could not be fully reconstructed. The downstream eligibility checks improve reproducibility, but they do not independently verify all source timestamps.

Other limitations include diagnosis code-based rather than imaging-confirmed ICH, incomplete differential counts, and the small eICU cohort. Hematoma volume, location, intraventricular extension, treatment intensity, infection, glucose-lowering therapy, and decisions to limit care were not harmonized, leaving substantial potential for residual confounding. Firth estimation cannot compensate for few events, and the patient-level bootstrap does not account for hospital clustering. Prediction CIs are conditional on the fitted development models and do not account for uncertainty from refitting them. Because the eICU cohort informed earlier exploratory work, it cannot provide untouched confirmatory validation. *p* values for the supplemental analyses were not adjusted for multiple testing. The sensitivity of the WBC count findings further emphasizes uncertainty about model form. Stronger prognostic or clinical use claims require confirmation in an independently assembled cohort with detailed imaging data.

## 5 Conclusion

Among patients with diagnosis code-defined ICH who were alive and hospitalized 24 h after ICU admission, joint WBC–glucose elevation was associated with subsequent in-hospital mortality in MIMIC-IV. The eICU cohort estimates were imprecise. The glucose level association in MIMIC-IV was nonlinear, whereas the WBC count curve depended on the spline specification; a stable U-shaped relationship was not established. Additional predictive value beyond the GCS score and the clinical baseline model remained uncertain. These findings describe prognostic associations and do not establish treatment thresholds or validate a standalone clinical tool.

## Data Availability

MIMIC-IV and eICU-CRD are available to credentialed PhysioNet users under their respective data-use agreements. Patient-level data cannot be redistributed. The accompanying reproducibility supplement contains downstream analysis code, aggregate results, prediction-model parameters and analysis definitions. It excludes source workbooks, patient identifiers and individual predictions. Full source-cohort reconstruction additionally requires the versioned database extraction queries and item mappings used during extraction. Further inquiries can be directed to the corresponding author.
