# Supplementary material for: Co-occurring leukocytosis and hyperglycemia identify patients at high risk of subsequent mortality after intracerebral hemorrhage: a 24-h landmark analysis in MIMIC-IV and eICU
Source: Front Neurol. 2026 Oct 30;17:1956229. doi: 10.3389/fneur.2026.1956229 (PMC13759353; doi:10.3389/fneur.2026.1956229)
Supplement: Supplementary file 1 [file Supplementary_file_1.pdf]

Supplementary Material

Co-occurring leukocytosis and hyperglycemia identify patients at high risk of subsequent mortality after intracerebral hemorrhage: a 24-hour landmark analysis in MIMIC-IV and eICU

Supplementary Table S1. Data provenance and reproducibility disclosure

Item	Implementation/evidence	Analytic implication
MIMIC-IV version	MIMIC-IV v3.1 was specified for the analysis; the harmonized workbook did not retain a release field.	The version and extraction date should match the source environment and repository record.
eICU version	eICU-CRD v2.0 was used for the cross-database evaluation.	The same database version was used for all reported eICU analyses.
Strict eICU cohort	264 patients and 42 post-landmark deaths under the identical ICD-9 431 or ICD-10 I61.x definition.	Broader code- or text-defined intracranial hemorrhage records were excluded from the primary cohort.
Hospital clustering	hospitalid was not retained in the harmonized eICU dataset.	Firth regression and patient-level bootstrap intervals were used; hospital-cluster resampling was not possible.
MIMIC-IV time fields	ICU outtime was discordant with hospital discharge or death for a subset of records.	Landmark eligibility used ICU admission plus hospital discharge/death timestamps; ICU outtime was not used.
GCS timing	Minimum GCS during ICU hours 0-24 was available; first GCS was not retained.	The GCS variable represents first-day neurological status and may incorporate deterioration, sedation, intubation, or treatment.
Reproducibility materials	Downstream workbook-level analysis code and an analytic data dictionary are available; source SQL and item mappings are required for full source-cohort reconstruction.	The harmonized analyses are reproducible from the analytic files, whereas complete source extraction requires the versioned SQL and mappings.

This table documents the provenance constraints of the harmonized analytic files and the corresponding analytic choices. It is not a substitute for the versioned SQL and item mappings required for complete source-cohort reconstruction.

Supplementary Table S2. Same-missing-information all-sample sensitivity

Model/comparison	N/events	AUROC	AUPRC	Brier
Log-continuous pair	264/42	0.835	0.426	0.114
Threshold category	264/42	0.839	0.439	0.113
Category minus log-continuous pair	Paired	0.004 (-0.011–0.019)	0.013 (-0.024–0.052)	-0.001 (-0.005–0.003)

WBC and glucose were imputed using MIMIC-IV medians, and both models received separate WBC- and glucose-missingness indicators so that the compared representations had identical access to missingness information.

Supplementary Table S3. Alternative threshold sensitivity

WBC cut	Glucose cut	AUROC	AUPRC	Brier	Delta AUROC vs log-continuous (95% CI)
10	140	0.831	0.478	0.118	0.003 (-0.014–0.019)
10	180	0.822	0.464	0.120	-0.006 (-0.027–0.013)
11	140	0.836	0.475	0.115	0.008 (-0.009–0.026)

WBC cut	Glucose cut	AUROC	AUPRC	Brier	Delta AUROC vs log-continuous (95% CI)
11	180	0.830	0.455	0.118	0.002 (-0.014–0.019)
12	140	0.839	0.475	0.116	0.011 (-0.008–0.031)
12	180	0.835	0.459	0.118	0.007 (-0.013–0.027)

Thresholds are shown in x10^9/L for WBC and mg/dL for glucose. These analyses were prespecified sensitivity combinations rather than data-driven searches for an optimal mortality cut point.

Supplementary Table S4. Internal nested validation and key calibration intervals

Model	Nested AUROC, mean (SD)	Nested AUPRC, mean (SD)	External calibration intercept (95% CI)	External calibration slope (95% CI)	Final C
Baseline + log WBC + log glucose	0.755 (0.033)	0.498 (0.069)	-0.38 (-1.035–0.340)	0.89 (0.536–1.422)	0.3
Baseline + flexible WBC/glucose splines	0.755 (0.029)	0.482 (0.062)	-0.48 (-1.187–0.354)	0.77 (0.404–1.373)	3
Baseline + threshold category	0.752 (0.030)	0.499 (0.060)	-0.50 (-1.189–0.345)	0.77 (0.403–1.409)	3

Internal estimates used repeated nested validation in MIMIC-IV. External calibration intervals were obtained from patient-level bootstrap samples of fixed eICU predictions.

Supplementary Table S5. Supplementary explanatory random-forest specification and ranking performance

Item	MIMIC-IV development	eICU evaluation	Interpretation
Population	1590 patients/325 events	229 patients/40 events	Same complete-marker 24-hour landmark population as the primary comparison
Model selection	24-combination five-fold grid; best mean AUPRC 0.557	No tuning	MIMIC-IV-only selection; 800-tree frozen refit
Ranking performance	Out-of-fold AUROC 0.819; AUPRC 0.542	AUROC 0.836 (0.767–0.895); AUPRC 0.518 (0.370–0.673)	Exploratory ranking only
Agreement with primary spline model	Not used for model selection	Spearman r=0.734; Pearson r=0.635	Moderate agreement; models are not interchangeable
SHAP target	Uncalibrated class-1 forest score	Not recomputed for external feature ranking	Not a calibrated clinical probability

The supplementary random forest used the complete-marker MIMIC-IV landmark cohort and 12 predictors: age, sex, minimum GCS, systolic and diastolic blood pressure, platelets, hemoglobin, creatinine, sodium, potassium, WBC, and glucose. Missing baseline values were median-imputed, with missingness indicators generated when applicable. The selected specification was refitted with 800 trees on all eligible MIMIC-IV patients and evaluated in eICU without further tuning. TreeSHAP values were calculated for the uncalibrated class-1 forest score and were not used to infer causality, biological interaction, or superiority of one WBC-glucose representation.

Supplementary Table S6. Bootstrap stability of grouped mean absolute SHAP values in MIMIC-IV

Feature	Mean absolute SHAP (95% CI)	Median rank (IQR)	Top-five frequency
Minimum GCS	0.177 (0.172–0.183)	1 (1–1)	100.0%
Age	0.055 (0.053–0.056)	2 (2–2)	100.0%
Glucose	0.052 (0.050–0.054)	3 (3–3)	100.0%
Platelets	0.030 (0.029–0.032)	4 (4–5)	100.0%
WBC	0.030 (0.029–0.031)	5 (4–5)	100.0%

Feature	Mean absolute SHAP (95% CI)	Median rank (IQR)	Top-five frequency
Hemoglobin	0.018 (0.017–0.019)	6 (6–6)	0.0%
Creatinine	0.017 (0.016–0.018)	7 (7–7)	0.0%
Sodium	0.016 (0.015–0.016)	8 (8–8)	0.0%
Diastolic BP	0.012 (0.011–0.012)	9 (9–9)	0.0%
Systolic BP	0.011 (0.011–0.011)	10 (10–10)	0.0%

Confidence intervals and rank frequencies are based on 500 patient-level resamples of the fixed MIMIC-IV SHAP attribution matrix. The random forest was not refitted in each resample.

Supplementary Table S7. Exploratory abnormality-burden, absolute-risk, and dual-versus-non-dual analyses

Dataset	0 abnormalities	1 abnormality	2 abnormalities	Unadjusted OR per additional abnormality (95% CI)	Absolute risk difference: dual vs normal (95% CI)	Risk ratio: dual vs non-dual (95% CI)
MIMIC-IV	12.3% (62/504)	19.7% (133/675)	31.6% (130/411)	1.82 (1.54-2.16); P<0.001	19.3 percentage points (14.0-24.6)	1.91 (1.58-2.32)
eICU	8.4% (7/83)	19.2% (19/99)	29.8% (14/47)	2.11 (1.31-3.40); P=0.002	21.4 percentage points (7.7-36.2)	2.09 (1.19-3.67)

The 1-abnormality group combined leukocytosis-only and hyperglycemia-only patients. Mortality proportions used Wilson intervals; absolute risk differences used Newcombe-Wilson intervals. The ordinal OR and dual-versus-non-dual risk ratio were unadjusted exploratory estimates and did not replace the adjusted four-category models.

Supplementary Figure S1. Grouped SHAP beeswarm plot for the frozen explanatory random forest in MIMIC-IV. Positive values increase the uncalibrated forest score; negative values decrease it.

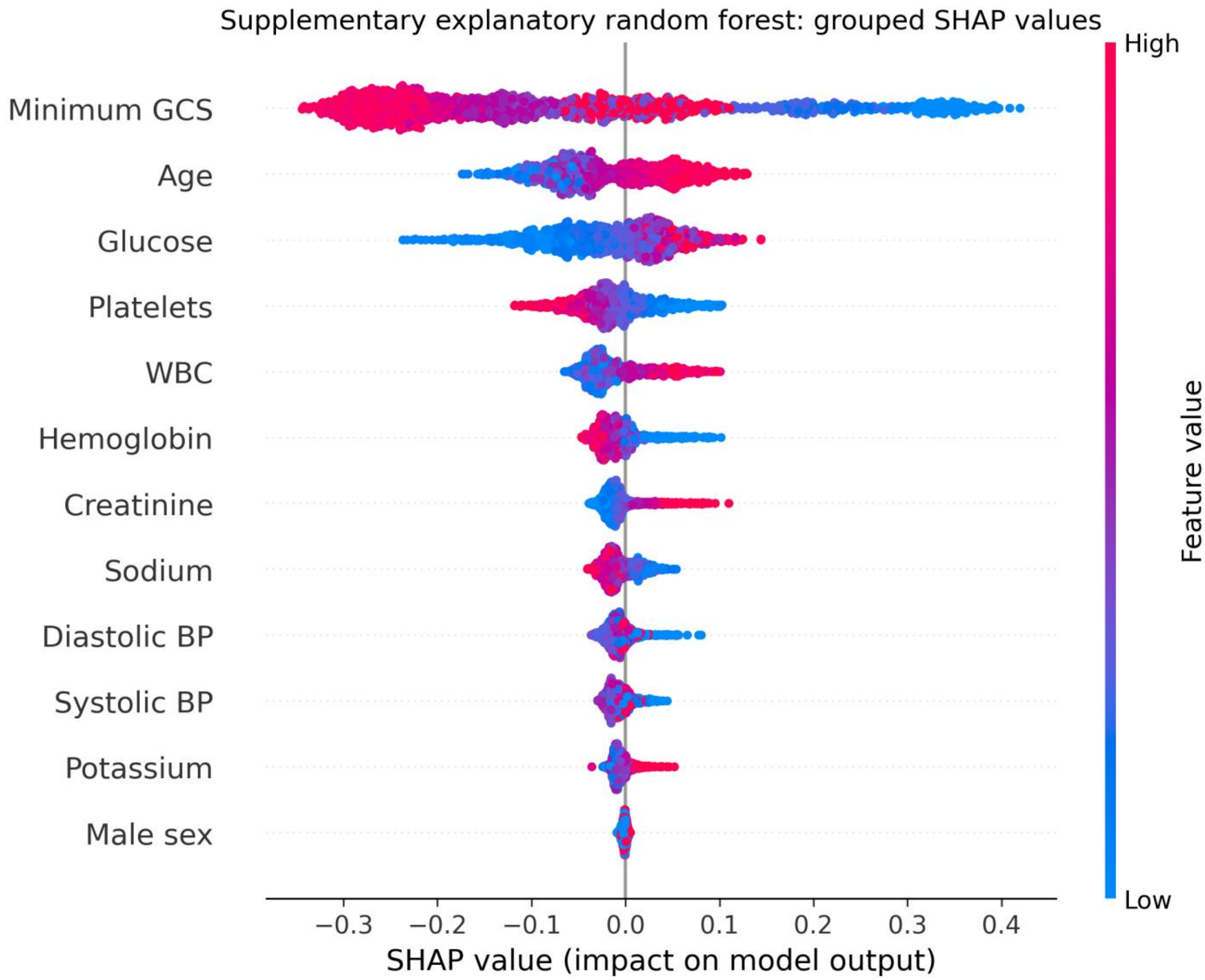

Supplementary Figure S2. Mean absolute grouped SHAP values with 95% intervals from 500 patient-level resamples of the fixed attribution matrix.

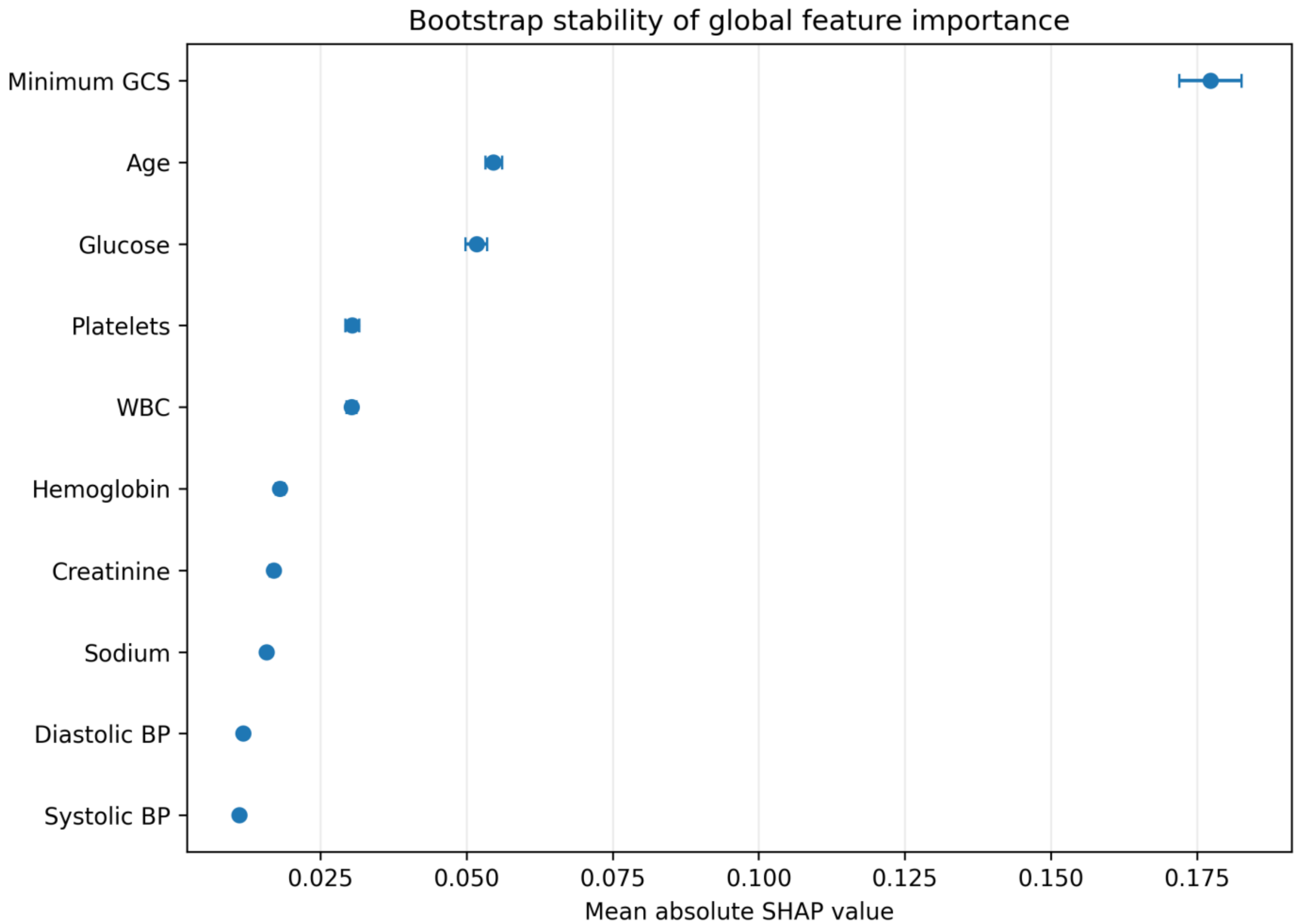

Supplementary Figure S3. WBC SHAP dependence plot, colored by glucose. The x-axis is restricted to the 1st-99th percentile for display; all observations contributed to fitting and SHAP computation.

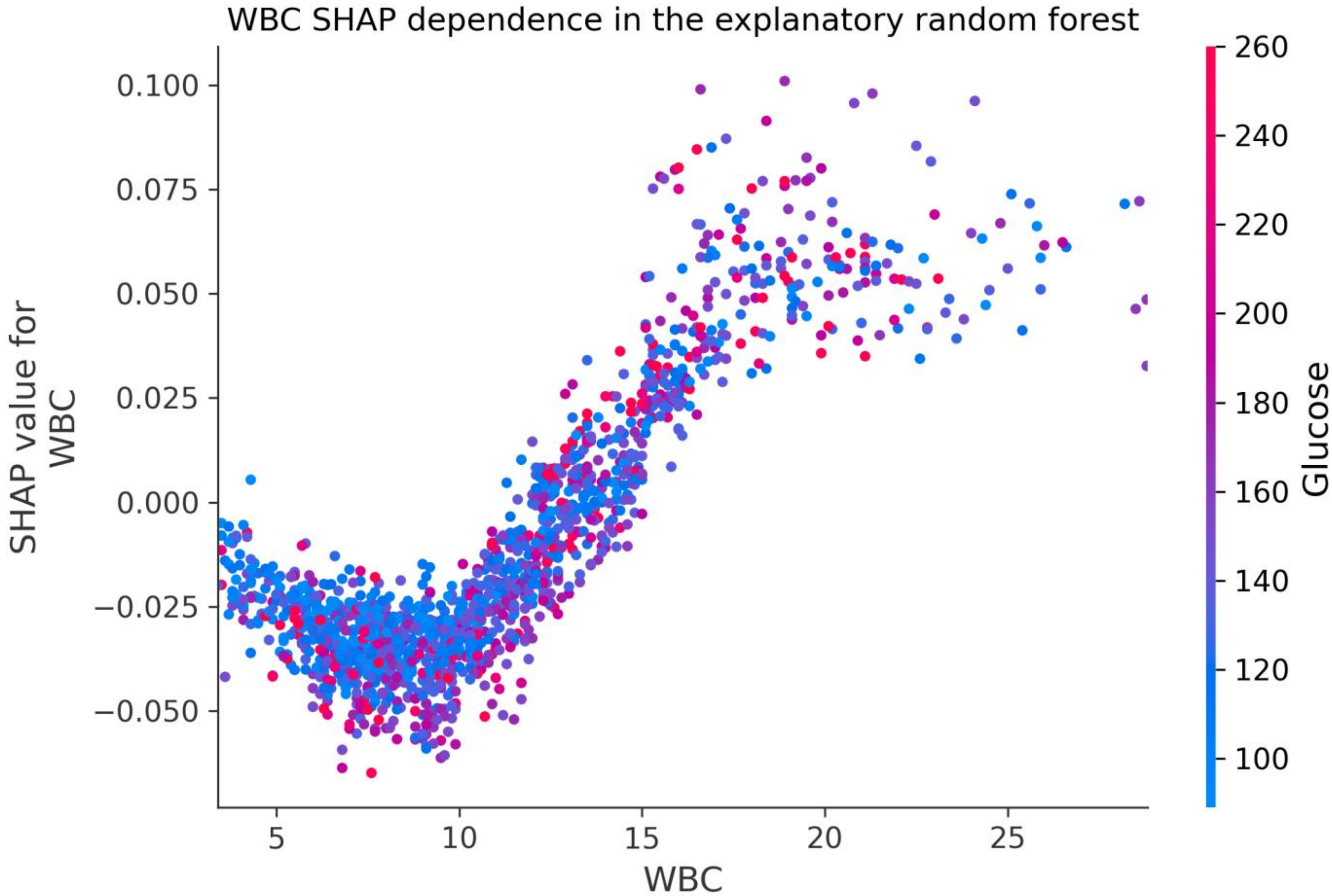

Supplementary Figure S4. Glucose SHAP dependence plot, colored by WBC. The x-axis is restricted to the 1st-99th percentile for display; all observations contributed to fitting and SHAP computation.

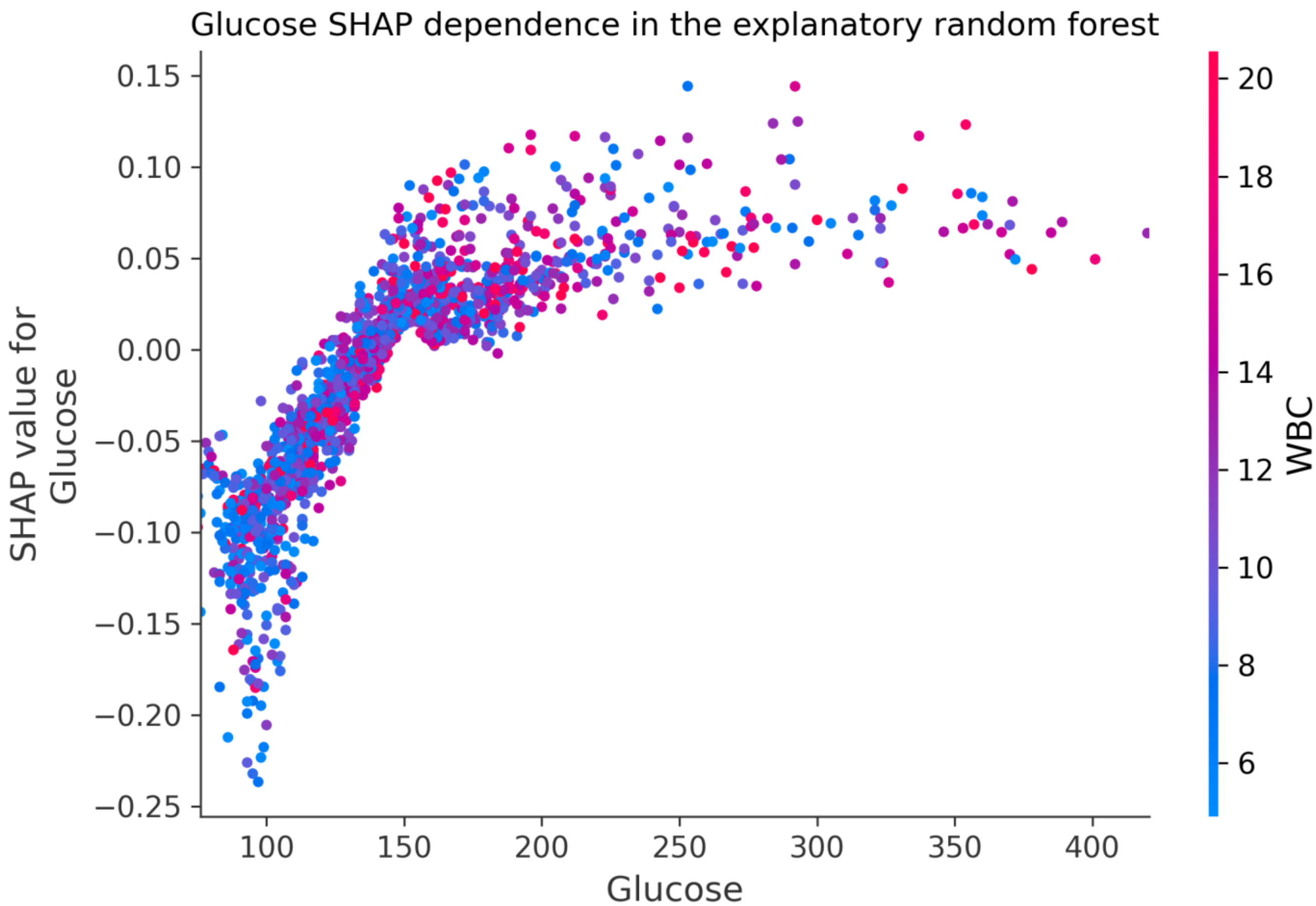

Supplementary Figure S5A. Representative explanation near the 10th percentile of the MIMIC-IV forest-score distribution.

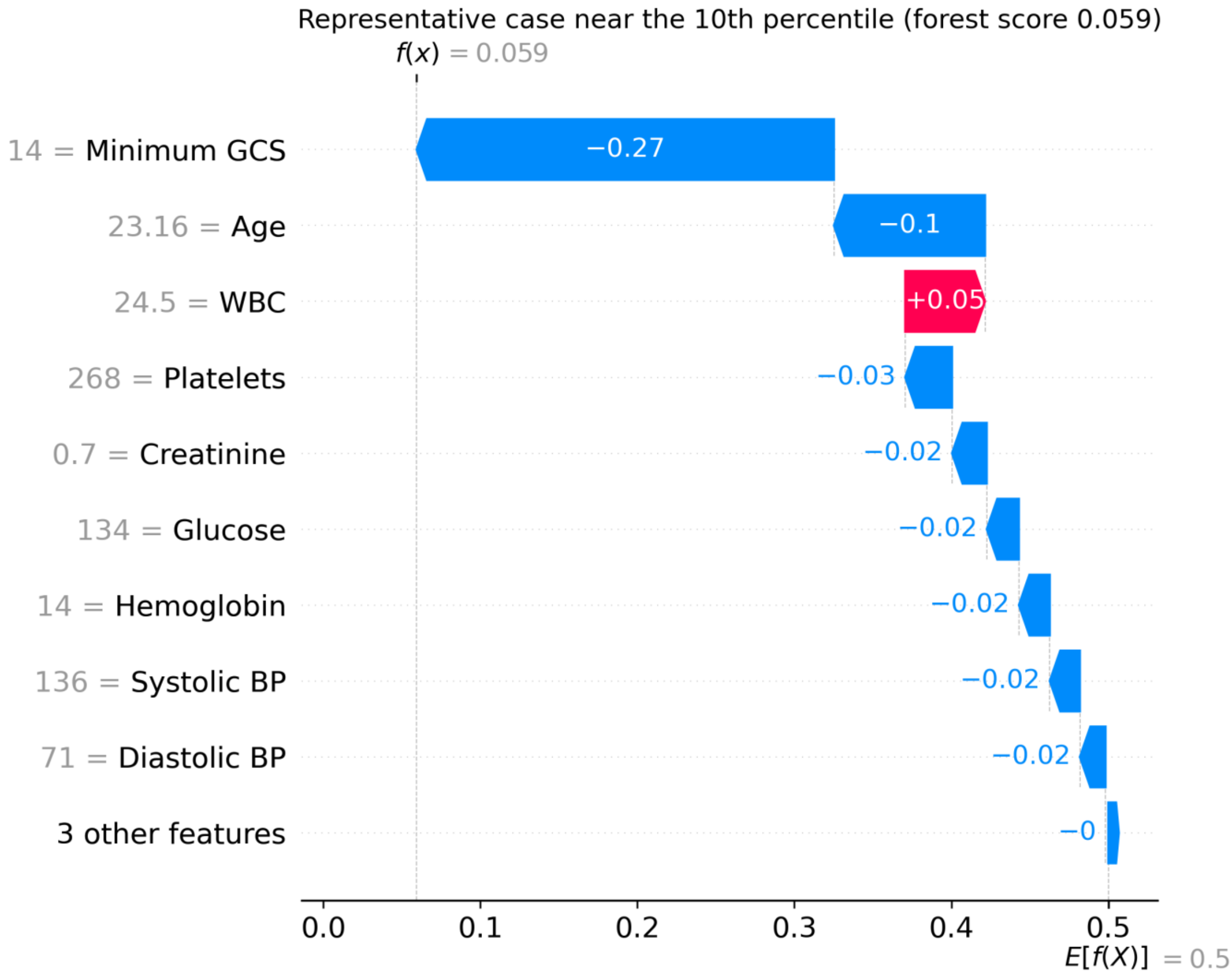

Supplementary Figure S5B. Representative explanation near the 50th percentile of the MIMIC-IV forest-score distribution.

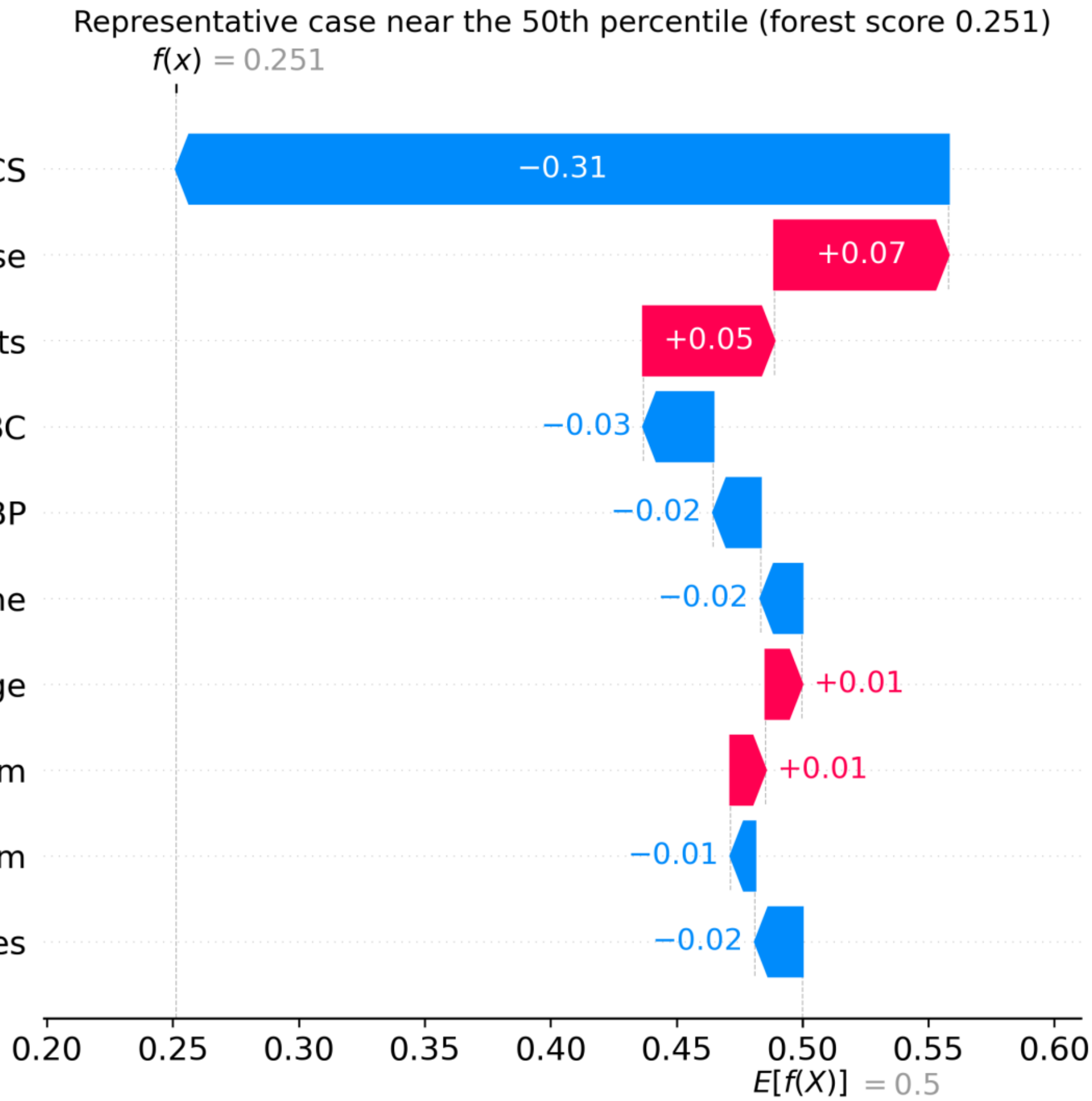

Supplementary Figure S5C. Representative explanation near the 90th percentile of the MIMIC-IV forest-score distribution.

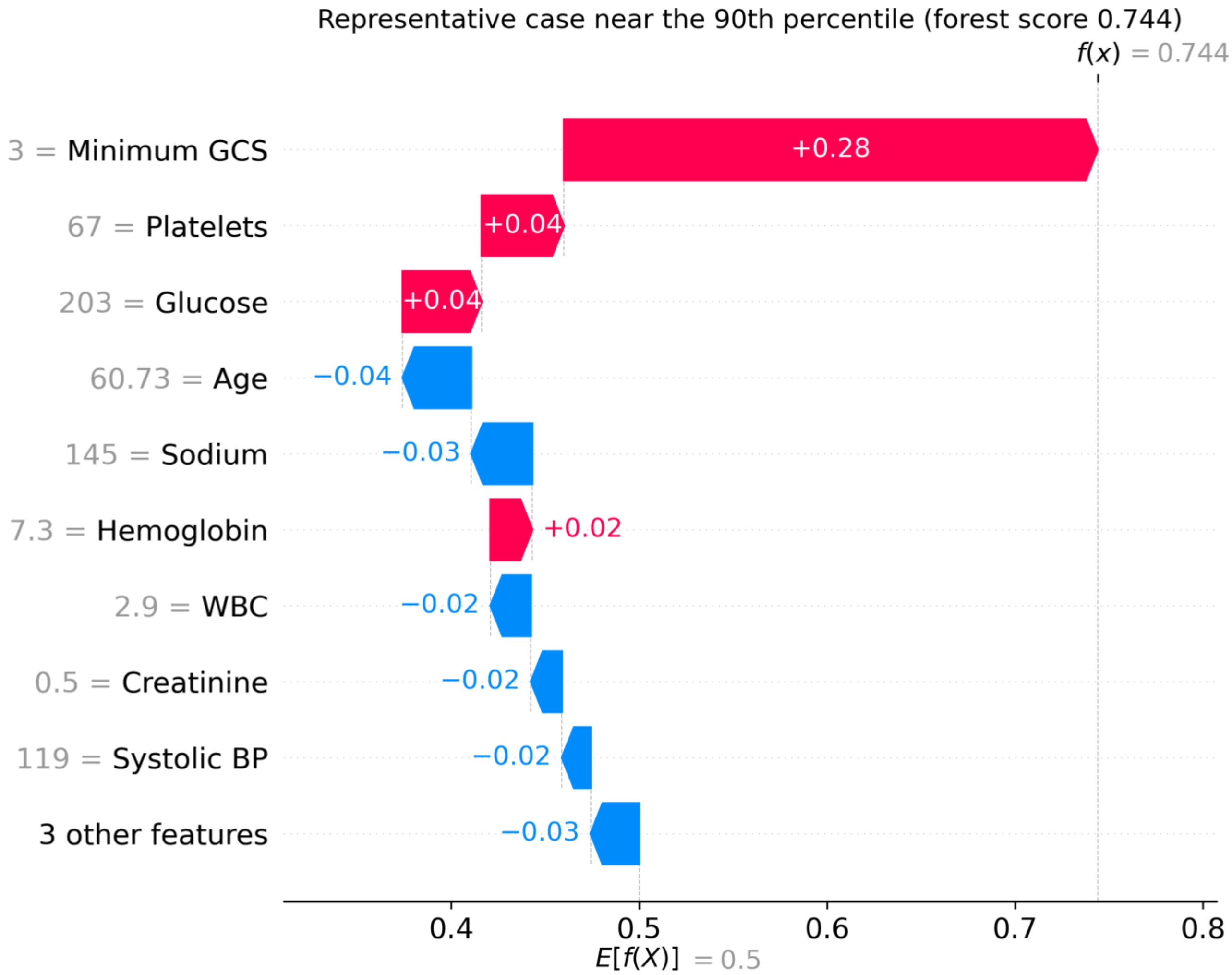

The three Supplementary Figure S5 examples were selected mechanically by forest-score percentile rather than by clinical appearance. They illustrate fitted model behavior and are not individual calibrated risk estimates.
