# Supplementary material for: Co-occurring leukocytosis and hyperglycemia identify patients at high risk of subsequent mortality after intracerebral hemorrhage: a 24-h landmark analysis in MIMIC-IV and eICU
Source: Front Neurol. 2026 Oct 30;17:1956229. doi: 10.3389/fneur.2026.1956229 (PMC13759353; doi:10.3389/fneur.2026.1956229)
Supplement: Supplementary file 2 [file Supplementary_file_2.docx]

Supplementary material

Co-occurring leukocytosis and hyperglycemia identify patients at high risk of subsequent mortality after intracerebral hemorrhage: a 24-hour landmark analysis in MIMIC-IV and eICU

Integrated revision | Supplementary Tables S1–S11 and Figures S1–S12

This supplement accompanies the revised manuscript. The primary analysis remains the cohort-specific four-group association model. Additional regression, nonlinear, lymphocyte, and prediction analyses are reported regardless of statistical significance. The machine-readable analysis files contain unrounded coefficients and aggregate results, but no patient-level data or individual predictions.

## Supplementary methods and table guide

Abbreviations: white blood cell count (WBC); absolute lymphocyte count (ALC); Glasgow Coma Scale (GCS); systolic/diastolic blood pressure (SBP/DBP); restricted cubic spline (RCS); odds ratio (OR); confidence interval (CI); likelihood ratio (LR); degrees of freedom (df); area under the receiver operating characteristic curve (AUROC); average precision (AP); standard deviation (SD). Database names follow the full manuscript title and definitions.

Association and prediction analyses use different samples and estimation methods. Association models require complete adjustment variables and use ordinary logistic regression in MIMIC-IV (1,589 patients/324 deaths) and Firth regression in eICU (221/37). Ridge prediction models include all patients with both markers measured (1,590/325 and 229/40), with missing baseline values imputed using training data. Association-model codes in Table S3 are distinct from the ridge prediction-model codes G0, G_log, G_rcs, G_cat, C0, and C_*.

For association models, RCS knots are calculated within each cohort; for prediction models, they are calculated within each training subset. The spline ORs describe adjusted associations, not predicted probabilities. Pointwise Wald confidence bands quantify uncertainty at individual values under the fitted model; they do not incorporate uncertainty about the choice of model form. When Firth-model coefficients are restricted for hypothesis testing, the Jeffreys penalty for the full design is retained. The aggregate analysis files document numerical checks of the solver and comparisons with an independently parameterized spline basis. These checks assess the implementation, not the accuracy of source-event records.

Scaled Brier = 1 − Brier/[p×(1−p)], where p is the observed mortality prevalence in the evaluation sample. It compares the model's probability error with a reference that assigns the same prevalence-based probability to every patient. Calibration-in-the-large assesses overall overprediction or underprediction with the prediction-logit slope fixed at one. A separate regression estimates the calibration intercept and slope together. Ideal values are zero for calibration-in-the-large and one for the slope. These diagnostics do not change the predictions being evaluated.

AP denotes average precision, not the trapezoidal area under the precision–recall curve. SDs across nested-validation repetitions describe variation caused by different data splits; repeated folds are not independent samples of patients. External bootstrap intervals hold the fitted models fixed and exclude uncertainty from repeating model development or accounting for hospital clustering. The two principal comparisons of added predictive value were specified before fitting this revision's prediction models. The remaining comparisons are exploratory, and new association P values are nominal, without adjustment for multiple testing.

Table guide: S1 provenance/selection/missingness; S2 GCS foundation; S3 alternative regressions; S4 overall tests/profile intervals; S5 nonlinear analyses; S6 reconstructed lymphocytes; S7 internal prediction; S8 calibration; S9 prediction sensitivities; S10 statistical interaction; S11 historical-to-revised prediction reconciliation.

# Table S1. Data provenance, cohort accounting and missingness

| **Cohort** | **Stage** | **Patients** | **Deaths** |
| --- | --- | --- | --- |
| MIMIC-IV | Input workbook | 1644 | 361 |
| MIMIC-IV | Strict ICD-9 431 / ICD-10 I61.x | 1637 | 361 |
| MIMIC-IV | 24-hour landmark | 1606 | 332 |
| MIMIC-IV | Complete WBC and glucose | 1590 | 325 |
| MIMIC-IV | Primary covariate-complete | 1589 | 324 |
| eICU | Input workbook | 2208 | 346 |
| eICU | Strict ICD-9 431 / ICD-10 I61.x | 264 | 42 |
| eICU | 24-hour landmark | 264 | 42 |
| eICU | Complete WBC and glucose | 229 | 40 |
| eICU | Primary covariate-complete | 221 | 37 |

Death counts in the input and strict-code rows are based on hospital-death flags. Landmark rows report subsequent in-hospital deaths among retained patients. The MIMIC-IV exclusions removed 29 records with hospital-death flags: 24 recorded early deaths and five eventual-death flags among seven other early-discharge records. These conflicting records are disclosed; the seven early discharges are not all classified as survivors.

| **Item** | **Implementation / limitation** |
| --- | --- |
| Database versions | MIMIC-IV v3.1 and eICU-CRD v2.0 were specified for extraction. The workbooks do not independently authenticate release metadata. |
| MIMIC time fields | Date fields parsed day/month/year. Eligibility requires hospital discharge and any recorded death after ICU admission +24 h. ICU outtime is not used because of discordance. |
| eICU time fields | Upstream extract states ICU stay >1 day. One rounded LOS of 1.00 is retained; exact offsets are absent, so the rounded number is not treated as an exact time. |
| Exposure and GCS timing | First ICU-day laboratory values and minimum first-day GCS are available. The analytic extracts lack row-level source-event linkage, same-draw timing and first GCS; a targeted raw-table review was limited to the order of magnitude and units of extreme WBC measurements. |
| Clustering | Hospital identifiers are not retained. eICU uncertainty is patient-level and does not account for hospital clustering. |
| Observed extreme WBC | Maximum 290.7×10⁹/L retained in primary models. Repeated source-table WBC measurements of the same order of magnitude were recorded in K/µL. The central 98% analysis is an influence check, not proof of input error. |
| Lymphocyte reconstruction | WBC×lymphocyte percentage/100; stored absolute counts are not used. Same-sample pairing cannot be confirmed. |
| Unmeasured variables | Imaging severity, treatments, infection, glycemic therapy and care-limitation decisions are not harmonized; residual confounding remains. |
| Reproducibility boundary | Downstream code and aggregate outputs are supplied. Source-level reconstruction additionally requires versioned SQL/item mappings. A targeted source-table quality check was performed, but no analytic variable or cohort was re-extracted or replaced. |

# Table S1 (continued). Predictor missingness

| **Variable** | **MIMIC-IV missing/N (%)** | **eICU missing/N (%)** |
| --- | --- | --- |
| Age | 0/1606 (0.0%) | 0/264 (0.0%) |
| Minimum GCS | 1/1606 (0.1%) | 6/264 (2.3%) |
| First SBP | 0/1606 (0.0%) | 4/264 (1.5%) |
| First DBP | 0/1606 (0.0%) | 4/264 (1.5%) |
| Platelets | 7/1606 (0.4%) | 38/264 (14.4%) |
| Hemoglobin | 8/1606 (0.5%) | 42/264 (15.9%) |
| Creatinine | 9/1606 (0.6%) | 25/264 (9.5%) |
| Anion gap | 15/1606 (0.9%) | 60/264 (22.7%) |
| Sodium | 15/1606 (0.9%) | 25/264 (9.5%) |
| Potassium | 15/1606 (0.9%) | 28/264 (10.6%) |
| WBC | 8/1606 (0.5%) | 35/264 (13.3%) |
| Glucose | 15/1606 (0.9%) | 18/264 (6.8%) |
| Sex | Not tabulated | Not tabulated |

GCS, Glasgow Coma Scale; SBP/DBP, systolic/diastolic blood pressure; WBC, white blood cell count. Denominators are the full landmark cohorts. Missingness counts for prediction variables include blood pressure values at or below zero treated as missing. Recorded diabetes is an adjustment variable in the MIMIC-IV association model but is not part of the common prediction baseline. Table S6 reports the availability of components needed to reconstruct ALC.

## Machine-learning analyses removed during revision

The random-forest/SHAP performance table, attribution plots, and correlations with the historical predictive spline were removed from this revised supplement. They are not used to support the rebuilt prediction results. Their removal focuses the manuscript on the clinical questions and does not constitute a negative test of an independent biomarker association. Historical ridge prediction values appear only in the comparison with revised values in Table S11.

# Table S2. GCS distributions and univariable associations

| **Cohort** | **Sample** | **Survivors: N; median (IQR)** | **Deaths: N; median (IQR)** |
| --- | --- | --- | --- |
| MIMIC-IV | Available GCS in landmark cohort | 1274; 13 (9–14) | 331; 7 (3.5–13) |
| MIMIC-IV | Common association sample | 1265; 13 (9–14) | 324; 7 (3–13) |
| eICU | Available GCS in landmark cohort | 218; 14 (10–15) | 40; 4 (3–7) |
| eICU | Common association sample | 184; 14 (9–15) | 37; 4 (3–7) |

| **Cohort** | **Sample** | **N/deaths** | **OR per 1-point lower GCS (95% CI)** | **P** |
| --- | --- | --- | --- | --- |
| MIMIC-IV | Available GCS | 1605/331 | 1.23 (1.19–1.27) | <0.001 |
| MIMIC-IV | Common association sample | 1589/324 | 1.23 (1.19–1.28) | <0.001 |
| eICU | Available GCS | 258/40 | 1.34 (1.23–1.47) | <0.001 |
| eICU | Common association sample | 221/37 | 1.32 (1.20–1.44) | <0.001 |

GCS, Glasgow Coma Scale; IQR, interquartile range; OR, odds ratio; CI, confidence interval. The available-GCS and common-sample analyses use different denominators. MIMIC-IV uses ordinary logistic regression, and eICU uses Firth regression. These univariable estimates describe associations; they do not measure improvement in predictive performance.

# Table S3. GCS-based alternative association models

Within each cohort, all models use the same complete-case sample: 1,589 patients/324 deaths in MIMIC-IV and 221 patients/37 deaths in eICU. Cells report ORs with Wald 95% CIs and P values. The aggregate coefficient file contains the intercept and all adjustment-variable coefficients.

| **Model** | **Variable / contrast** | **MIMIC-IV** | **eICU** |
| --- | --- | --- | --- |
| G0 | Minimum GCS, per 1-point decrease | 1.23 (1.19–1.28); P<0.001 | 1.32 (1.20–1.44); P<0.001 |
| G1 | Minimum GCS, per 1-point decrease | 1.22 (1.18–1.27); P<0.001 | 1.29 (1.17–1.41); P<0.001 |
|  | WBC, per 5 ×10⁹/L increase | 1.04 (0.99–1.09); P=0.110 | 1.49 (0.88–2.54); P=0.140 |
|  | Glucose, per 50-mg/dL increase | 1.27 (1.16–1.39); P<0.001 | 1.21 (0.86–1.71); P=0.279 |
| G2 | Minimum GCS, per 1-point decrease | 1.22 (1.18–1.26); P<0.001 | 1.30 (1.18–1.42); P<0.001 |
|  | WBC >10 vs ≤10 ×10⁹/L | 1.32 (1.01–1.73); P=0.041 | 1.15 (0.49–2.68); P=0.752 |
|  | Glucose >140 vs ≤140 mg/dL | 1.84 (1.41–2.41); P<0.001 | 1.71 (0.76–3.84); P=0.194 |
| G3 | Minimum GCS, per 1-point decrease | 1.22 (1.18–1.26); P<0.001 | 1.29 (1.18–1.42); P<0.001 |
|  | WBC elevation only vs neither elevated | 1.35 (0.92–1.98); P=0.128 | 1.12 (0.38–3.37); P=0.835 |
|  | Glucose elevation only vs neither elevated | 1.88 (1.25–2.83); P=0.002 | 1.69 (0.42–6.74); P=0.458 |
|  | Both markers elevated vs neither elevated | 2.45 (1.72–3.49); P<0.001 | 1.94 (0.64–5.89); P=0.241 |
| G4 | Minimum GCS, per 1-point decrease | 1.22 (1.18–1.26); P<0.001 | 1.30 (1.18–1.42); P<0.001 |
|  | One elevated marker vs none | 1.55 (1.11–2.18); P=0.011 | 1.23 (0.44–3.46); P=0.693 |
|  | Two elevated markers vs none | 2.45 (1.71–3.49); P<0.001 | 1.93 (0.64–5.88); P=0.245 |
| G5 | Minimum GCS, per 1-point decrease | 1.22 (1.18–1.26); P<0.001 | 1.30 (1.18–1.43); P<0.001 |
|  | Per additional elevated marker (ordinal assumption) | 1.57 (1.31–1.87); P<0.001 | 1.41 (0.81–2.47); P=0.227 |

G0: GCS alone. G1: continuous pair. G2: two elevation indicators without product. G3: four-category joint status. G4: three-level count factor. G5: ordinal count. Continuous ORs assume linear log-odds terms. Ordinal counts assume equal log-odds increments. Alternative parameterizations are not independent validations. WBC, white blood cell count; GCS, Glasgow Coma Scale; SBP, systolic blood pressure; OR, odds ratio; CI, confidence interval.

# Table S3. Alternative representations with original clinical adjustment

Within each cohort, all models use the same complete-case sample: 1,589 patients/324 deaths in MIMIC-IV and 221 patients/37 deaths in eICU. Cells report ORs with Wald 95% CIs and P values. The aggregate coefficient file contains the intercept and all adjustment-variable coefficients.

| **Model** | **Variable / contrast** | **MIMIC-IV** | **eICU** |
| --- | --- | --- | --- |
| F1 | WBC, per 5 ×10⁹/L increase | 1.05 (0.99–1.10); P=0.106 | 1.50 (0.85–2.62); P=0.161 |
|  | Glucose, per 50-mg/dL increase | 1.27 (1.15–1.41); P<0.001 | 1.39 (0.98–1.96); P=0.065 |
| F2 | WBC >10 vs ≤10 ×10⁹/L | 1.67 (1.26–2.23); P<0.001 | 1.27 (0.52–3.10); P=0.603 |
|  | Glucose >140 vs ≤140 mg/dL | 1.78 (1.34–2.38); P<0.001 | 2.33 (0.97–5.57); P=0.057 |
| F3 | WBC elevation only vs neither elevated | 1.67 (1.12–2.49); P=0.012 | 1.95 (0.58–6.58); P=0.282 |
|  | Glucose elevation only vs neither elevated | 1.78 (1.15–2.75); P=0.009 | 4.64 (1.02–21.03); P=0.047 |
|  | Both markers elevated vs neither elevated | 2.98 (2.04–4.36); P<0.001 | 3.34 (0.97–11.54); P=0.057 |
| F4 | One elevated marker vs none | 1.72 (1.20–2.44); P=0.003 | 2.35 (0.74–7.46); P=0.149 |
|  | Two elevated markers vs none | 2.98 (2.04–4.36); P<0.001 | 3.23 (0.94–11.08); P=0.062 |
| F5 | Per additional elevated marker (ordinal assumption) | 1.73 (1.43–2.08); P<0.001 | 1.74 (0.96–3.15); P=0.066 |

F1: continuous pair. F2: two binary elevation indicators. F3: primary four-category model. F4: three-level count factor. F5: exploratory ordinal count. Original adjustment: MIMIC-IV age, sex, GCS, SBP, platelets, creatinine, diabetes; eICU age, sex, GCS, SBP. Continuous ORs assume linear log-odds terms. Ordinal counts assume equal log-odds increments. Alternative parameterizations are not independent validations. WBC, white blood cell count; GCS, Glasgow Coma Scale; SBP, systolic blood pressure; OR, odds ratio; CI, confidence interval.

# Table S4. Overall exposure tests and eICU profile inference

| **Association model** | **Exposure df** | **MIMIC-IV LR P** | **eICU penalized LR P** | **eICU Wald P** |
| --- | --- | --- | --- | --- |
| G1 | 2 | <0.001 | 0.166 | 0.160 |
| G2 | 2 | <0.001 | 0.387 | 0.388 |
| G3 | 3 | <0.001 | 0.589 | 0.592 |
| G4 | 2 | <0.001 | 0.460 | 0.461 |
| G5 | 1 | <0.001 | 0.223 | 0.227 |
| F1 | 2 | <0.001 | 0.056 | 0.050 |
| F2 | 2 | <0.001 | 0.120 | 0.123 |
| F3 | 3 | <0.001 | 0.141 | 0.156 |
| F4 | 2 | <0.001 | 0.151 | 0.170 |
| F5 | 1 | <0.001 | 0.063 | 0.066 |

LR, likelihood ratio; df, degrees of freedom. Table S3 defines the models. For eICU, the full-design Jeffreys penalty is retained when coefficients are restricted for testing. An overall exposure test and an individual category comparison address different hypotheses; neither measures improvement in prediction.

## Primary eICU category contrasts: all profile-penalized estimates

| **Contrast vs neither elevated** | **OR** | **Profile 95% CI** | **Profile P** |
| --- | --- | --- | --- |
| WBC elevation only | 1.9497 | 0.5912–6.9868 | 0.2757 |
| Glucose elevation only | 4.6374 | 1.0162–21.8226 | 0.0477 |
| Both elevated | 3.3409 | 0.9980–12.2046 | 0.0504 |

For both markers elevated, the lower profile CI limit is 0.9980 and P=0.0504. Rounding must not be interpreted as a lower limit above 1 or P<0.05. Main Table 3 retains Wald intervals and tests for consistency with the submitted analysis. Profile-penalized results are provided for all three comparisons, not only a selected comparison.

## Unadjusted Table 2 checks

Pearson four-category P: MIMIC-IV 4.1091×10⁻¹²; eICU 0.0198758. Three-level count P: MIMIC-IV 4.1841×10⁻¹²; eICU 0.0072591. eICU four-category Monte Carlo P=0.01952, simulation SE=0.00044; 100,000 draws with fixed margins, seed 20260922. No selected pairwise post-hoc tests were added.

# Table S5. Nonlinear association tests, knots and low-end support

| **Cohort** | **Specification** | **N/deaths** | **Marker** | **Overall P** | **Nonlinear P** |
| --- | --- | --- | --- | --- | --- |
| MIMIC-IV | Primary: 3 knots | 1589/324 | WBC | 0.131 | 0.230 |
| MIMIC-IV | Primary: 3 knots | 1589/324 | Glucose | <0.001 | 0.005 |
| MIMIC-IV | Sensitivity: 4 knots | 1589/324 | WBC | <0.001 | <0.001 |
| MIMIC-IV | Sensitivity: 4 knots | 1589/324 | Glucose | <0.001 | 0.013 |
| MIMIC-IV | Sensitivity: both markers within 1st–99th percentiles | 1528/304 | WBC | <0.001 | 0.222 |
| MIMIC-IV | Sensitivity: both markers within 1st–99th percentiles | 1528/304 | Glucose | <0.001 | 0.024 |
| eICU | Primary: 3 knots | 221/37 | WBC | 0.071 | 0.064 |
| eICU | Primary: 3 knots | 221/37 | Glucose | 0.178 | 0.825 |

Degrees of freedom for overall/nonlinearity tests are 2/1 for three knots and 3/2 for four knots. MIMIC-IV uses ordinary LR tests, and eICU uses full-design penalized LR tests. These nominal association tests do not, by themselves, establish a U-shaped relationship. WBC, white blood cell count; LR, likelihood ratio.

| **Cohort** | **Specification** | **Marker** | **Knots** |
| --- | --- | --- | --- |
| MIMIC-IV | Primary: 3 knots | wbc | 5.9; 10.3; 17.3 |
| MIMIC-IV | Primary: 3 knots | glucose | 95; 134; 211.2 |
| MIMIC-IV | Sensitivity: 4 knots | wbc | 4.9; 8.7; 12.2; 20.56 |
| MIMIC-IV | Sensitivity: 4 knots | glucose | 89; 119; 150; 260 |
| MIMIC-IV | Sensitivity: both markers within 1st–99th percentiles | wbc | 5.9; 10.3; 17.3 |
| MIMIC-IV | Sensitivity: both markers within 1st–99th percentiles | glucose | 95; 134; 211.2 |
| eICU | Primary: 3 knots | wbc | 6.6; 10.2; 15.2 |
| eICU | Primary: 3 knots | glucose | 96; 124; 208 |

Reference values are WBC=10×10⁹/L and glucose=140 mg/dL. The central 98% sensitivity model retains the original knots but restricts both markers to their original primary-sample 1st–99th percentile intervals: WBC 3.4–28.836×10⁹/L and glucose 76–420.84 mg/dL. It excludes 61 patients/20 deaths. All observations remain in the primary models. These specifications were defined for the revision analysis; they were not prespecified in the original study.

| **Low range in common association sample** | **MIMIC-IV N/deaths** | **eICU N/deaths** |
| --- | --- | --- |
| WBC <4×10⁹/L | 38/10 | 0/0 |
| Glucose <70 mg/dL | 9/1 | 2/0 |
| Glucose <60 mg/dL | 5/1 | 1/0 |

The glucose ranges overlap and should not be treated as independent groups. Low-glucose observations are very sparse, and eICU contains no observations in the leukopenic range. These limitations prevent firm conclusions about risk at low values. A nonsignificant nonlinearity test does not establish linearity or show that low values are safe.

# Table S6. Exploratory reconstructed lymphopenia

ALC was reconstructed as WBC×lymphocyte percentage/100, in ×10⁹/L. Lymphopenia was defined as ALC<1.0. ALC remained missing if either component was missing. We could not verify that the components came from the same blood sample and did not substitute the stored absolute-count values.

| **Cohort** | **ALC category** | **Deaths/N** | **Mortality % (95% CI)** |
| --- | --- | --- | --- |
| MIMIC-IV | ALC >=1.0 | 112/641 | 17.5 (14.7–20.6) |
| MIMIC-IV | ALC <1.0 | 110/437 | 25.2 (21.3–29.4) |
| eICU | ALC >=1.0 | 10/78 | 12.8 (7.1–22.0) |
| eICU | ALC <1.0 | 7/47 | 14.9 (7.4–27.7) |

ALC was available for 1,078/1,606 MIMIC-IV patients (67.1%) and 125/264 eICU patients (47.3%). Mortality CIs are Wilson intervals. These descriptive samples include all patients with reconstructed ALC and differ from the samples used in adjusted models.

| **Cohort** | **Model** | **N/deaths** | **OR (95% CI)** | **Wald P** |
| --- | --- | --- | --- | --- |
| MIMIC-IV | Unadjusted; same primary sample | 1076/222 | 1.59 (1.18–2.14) | 0.002 |
| MIMIC-IV | Primary clinical adjustment | 1076/222 | 1.54 (1.12–2.14) | 0.009 |
| eICU | Unadjusted; same primary sample | 122/16 | 1.30 (0.46–3.68) | 0.626 |
| eICU | Primary clinical adjustment | 122/16 | 0.80 (0.24–2.70) | 0.718 |
| eICU | ALC original adjustment sensitivity | 120/16 | 1.33 (0.31–5.67) | 0.702 |

The primary MIMIC-IV model adjusts for age, sex, GCS, SBP, platelets, creatinine, and diabetes. The primary eICU model adjusts for age and GCS; its sensitivity model also includes sex and SBP. eICU uses Firth estimation. Neither analysis additionally adjusts for total WBC or glucose, so the results do not establish whether the WBC association is independent of lymphocyte composition.

## eICU profile-penalized sensitivity

| **Model** | **OR** | **Profile 95% CI** | **Profile P** |
| --- | --- | --- | --- |
| Unadjusted; same primary sample | 1.30 | 0.45–3.65 | 0.624 |
| Primary clinical adjustment | 0.80 | 0.23–2.65 | 0.715 |
| ALC original adjustment sensitivity | 1.33 | 0.30–6.10 | 0.703 |

# Table S6 (continued). Availability and selection limits

| **Cohort** | **ALC status** | **N/deaths** | **Median age** | **Median GCS** |
| --- | --- | --- | --- | --- |
| MIMIC-IV | Available | 1078/222 | 68.7 | 11.0 |
| MIMIC-IV | Unavailable | 528/110 | 70.0 | 12.0 |
| eICU | Available | 125/17 | 64.0 | 14.0 |
| eICU | Unavailable | 139/25 | 64.0 | 13.0 |

ALC, absolute lymphocyte count; GCS, Glasgow Coma Scale; SBP, systolic blood pressure; WBC, white blood cell count; OR, odds ratio; CI, confidence interval. Missingness was not assumed to be random. Among patients without reconstructed ALC, mortality counts were 110/528 in MIMIC-IV and 25/139 in eICU. The eICU adjusted sample contains only 16 deaths; results from both the simpler primary model and the four-variable sensitivity model are reported. No ALC prediction model was developed, and no additional source data were extracted.

This exploratory analysis addresses leukocyte composition. It does not test mediation, validate the reconstructed measurement, or establish infection as a cause of mortality. Morotti et al. used a window relative to ICH onset and assessed 90-day outcomes, whereas this study uses an ICU landmark and subsequent in-hospital mortality. The primary four-group samples and estimates are unchanged.

# Table S7. Internal nested prediction evaluation

MIMIC-IV: 1,590 patients/325 deaths. Internal evaluation used three repetitions of five-fold outer cross-validation with three-fold inner tuning. All ten models used identical data splits.

| **Code** | **Model** | **AUROC mean (SD)** | **AP mean (SD)** | **Brier mean (SD)** |
| --- | --- | --- | --- | --- |
| G0 | GCS baseline | 0.675 (0.0002) | 0.458 (0.0015) | 0.1421 (0.0001) |
| G_log | GCS + log pair | 0.722 (0.0023) | 0.469 (0.0061) | 0.1394 (0.0005) |
| G_rcs | GCS + RCS pair | 0.724 (0.0012) | 0.463 (0.0007) | 0.1397 (0.0001) |
| G_cat | GCS + joint category | 0.712 (0.0026) | 0.477 (0.0022) | 0.1406 (0.0003) |
| C0 | Clinical baseline | 0.732 (0.0020) | 0.477 (0.0033) | 0.1380 (0.0001) |
| C_wbc | Clinical + log WBC | 0.739 (0.0012) | 0.481 (0.0066) | 0.1375 (0.0003) |
| C_glucose | Clinical + log glucose | 0.751 (0.0011) | 0.482 (0.0059) | 0.1362 (0.0005) |
| C_log | Clinical + log pair | 0.754 (0.0021) | 0.482 (0.0066) | 0.1356 (0.0005) |
| C_rcs | Clinical + RCS pair | 0.752 (0.0017) | 0.471 (0.0085) | 0.1365 (0.0007) |
| C_cat | Clinical + joint category | 0.750 (0.0011) | 0.488 (0.0078) | 0.1363 (0.0005) |

For each repetition, metrics are calculated from all held-out patient predictions and then summarized across the three repetitions. The SD describes variation between splits; it is not a standard error or CI. The aggregate files contain results for all 15 outer folds and all repetitions, selected tuning parameters, and fold hashes. Repeated predictions and folds are not treated as independent patients.

## Principal paired internal increments in each repetition

| **Comparison** | **Repeat** | **ΔAUROC** | **ΔAP** | **ΔBrier** |
| --- | --- | --- | --- | --- |
| G_cat − G0 | 1 | 0.039 | 0.016 | -0.0016 |
| G_cat − G0 | 2 | 0.034 | 0.222 | -0.0013 |
| G_cat − G0 | 3 | 0.038 | 0.022 | -0.0014 |
| C_cat − C0 | 1 | 0.021 | 0.016 | -0.0022 |
| C_cat − C0 | 2 | 0.015 | 0.007 | -0.0013 |
| C_cat − C0 | 3 | 0.018 | 0.010 | -0.0016 |

GCS, Glasgow Coma Scale; AP, average precision; AUROC, area under the receiver operating characteristic curve; SD, standard deviation. G0/G_* use GCS; C0/C_* use the common clinical baseline. Log pair means natural-log WBC and glucose; RCS means restricted cubic spline pair; category means the four-level joint status. Internal gain is not proof of gain in a different clinical population.

# Table S7 (continued). Final development tuning and model sizes

| **Model** | **Sample** | **Development N/deaths** | **Evaluation N/deaths** | **Selected C** | **Features** |
| --- | --- | --- | --- | --- | --- |
| G0 | Complete markers | 1590/325 | 229/40 | 0.03 | 2 |
| G_log | Complete markers | 1590/325 | 229/40 | 0.03 | 4 |
| G_rcs | Complete markers | 1590/325 | 229/40 | 1.0 | 6 |
| G_cat | Complete markers | 1590/325 | 229/40 | 0.03 | 6 |
| C0 | Complete markers | 1590/325 | 229/40 | 0.03 | 11 |
| C_wbc | Complete markers | 1590/325 | 229/40 | 1.0 | 12 |
| C_glucose | Complete markers | 1590/325 | 229/40 | 0.3 | 12 |
| C_log | Complete markers | 1590/325 | 229/40 | 1.0 | 13 |
| C_rcs | Complete markers | 1590/325 | 229/40 | 0.3 | 15 |
| C_cat | Complete markers | 1590/325 | 229/40 | 0.1 | 15 |
| All_log | All landmark | 1606/332 | 264/42 | 3.0 | 20 |
| All_cat | All landmark | 1606/332 | 264/42 | 0.03 | 22 |
| T_10_180 | Complete markers | 1590/325 | 229/40 | 0.03 | 15 |
| T_11_140 | Complete markers | 1590/325 | 229/40 | 0.03 | 15 |
| T_11_180 | Complete markers | 1590/325 | 229/40 | 0.03 | 15 |
| T_12_140 | Complete markers | 1590/325 | 229/40 | 0.03 | 15 |
| T_12_180 | Complete markers | 1590/325 | 229/40 | 0.03 | 15 |
| AG_log | Complete markers | 1590/325 | 229/40 | 0.3 | 14 |
| AG_cat | Complete markers | 1590/325 | 229/40 | 0.03 | 16 |

C is the inverse ridge-penalty strength, selected in MIMIC-IV alone by mean average precision. “Features” counts transformed model columns, not patients or distinct original clinical measurements. The C grid is 0.03, 0.1, 0.3, 1, 3. The file frozen_model_parameters.json records preprocessing, four-indicator category coding, knots, coefficients, and training medians and scales. All/AG/T codes identify sensitivity models defined in Table S9. Intercepts, binary indicators, and missingness indicators are distinguished from standardized continuous features.

# Table S8. eICU calibration diagnostics

| **Model** | **Calibration-in-the-large (95% CI)** | **Joint intercept (95% CI)** | **Calibration slope (95% CI)** |
| --- | --- | --- | --- |
| G0 | -0.226 (-0.597 to 0.094) | 0.265 (-0.334 to 0.879) | 1.477 (1.023 to 2.120) |
| G_log | -0.203 (-0.589 to 0.128) | 0.122 (-0.477 to 0.762) | 1.313 (0.906 to 1.851) |
| G_rcs | -0.211 (-0.610 to 0.129) | -0.007 (-0.560 to 0.567) | 1.209 (0.853 to 1.674) |
| G_cat | -0.195 (-0.570 to 0.129) | 0.252 (-0.340 to 0.860) | 1.428 (0.995 to 2.034) |
| C0 | -0.144 (-0.522 to 0.195) | 0.201 (-0.386 to 0.848) | 1.308 (0.904 to 1.868) |
| C_wbc | -0.153 (-0.535 to 0.198) | 0.062 (-0.471 to 0.641) | 1.206 (0.860 to 1.654) |
| C_glucose | -0.101 (-0.510 to 0.258) | -0.006 (-0.543 to 0.622) | 1.086 (0.767 to 1.494) |
| C_log | -0.111 (-0.509 to 0.243) | 0.023 (-0.503 to 0.646) | 1.125 (0.823 to 1.528) |
| C_rcs | -0.108 (-0.506 to 0.247) | 0.010 (-0.514 to 0.629) | 1.108 (0.792 to 1.528) |
| C_cat | -0.108 (-0.493 to 0.243) | 0.055 (-0.487 to 0.701) | 1.149 (0.813 to 1.636) |

Every model is evaluated in the same 229 patients/40 deaths. Calibration-in-the-large fixes the prediction-logit slope at one. A separate regression estimates the joint intercept and slope, whose ideal values are zero and one, respectively. These diagnostics do not update or recalibrate the evaluated probabilities. Intervals are based on patient resampling conditional on the fitted development models; hospital clustering and uncertainty from model refitting are not included. Table S7 defines the models. CI, confidence interval.

## Scaled probability error

| **Model** | **Scaled Brier (95% CI)** |
| --- | --- |
| G0 | 0.209 (0.092–0.300) |
| G_log | 0.197 (0.071–0.292) |
| G_rcs | 0.210 (0.074–0.314) |
| G_cat | 0.216 (0.096–0.307) |
| C0 | 0.183 (0.069–0.272) |
| C_wbc | 0.200 (0.073–0.300) |
| C_glucose | 0.178 (0.046–0.276) |
| C_log | 0.189 (0.058–0.288) |
| C_rcs | 0.183 (0.052–0.284) |
| C_cat | 0.194 (0.067–0.295) |

Scaled Brier uses a prevalence-only reference: 1 − Brier/[p(1−p)]. Higher values indicate less prediction error relative to this reference but do not independently establish clinical usefulness.

# Table S9. Predictive sensitivity analyses

The original sensitivity analyses of alternative thresholds, anion gap, and the full landmark samples were repeated using the explicitly specified revision workflow. They were not used to select an optimal clinical cutoff.

| **WBC/glucose cutoffs** | **AUROC** | **AP** | **Brier** | **ΔAUROC vs log pair (95% CI)** |
| --- | --- | --- | --- | --- |
| 10 / 140 | 0.811 | 0.470 | 0.1162 | -0.007 (-0.033 to 0.016) |
| 10 / 180 | 0.804 | 0.450 | 0.1178 | -0.013 (-0.039 to 0.010) |
| 11 / 140 | 0.814 | 0.475 | 0.1157 | -0.004 (-0.031 to 0.020) |
| 11 / 180 | 0.811 | 0.476 | 0.1164 | -0.006 (-0.031 to 0.017) |
| 12 / 140 | 0.817 | 0.475 | 0.1153 | 0.000 (-0.027 to 0.024) |
| 12 / 180 | 0.815 | 0.475 | 0.1160 | -0.002 (-0.027 to 0.022) |

All rows use the eICU sample of 229/40 patients/deaths. WBC is expressed in ×10⁹/L and glucose in mg/dL. Every model includes the clinical baseline, with tuning performed in the training data only. eICU outcomes were not used to select thresholds.

| **Sensitivity** | **N/deaths** | **Log/category AUROC** | **ΔAUROC category−log** | **ΔBrier category−log** |
| --- | --- | --- | --- | --- |
| All landmark; matched marker missingness | 264/42 | 0.847 / 0.817 | -0.030 (-0.075 to 0.007) | 0.0043 (-0.0020 to 0.0111) |
| Anion gap restored | 229/40 | 0.815 / 0.816 | 0.001 (-0.018 to 0.019) | 0.0003 (-0.0037 to 0.0045) |

In the all-landmark analysis, missing marker values are imputed using raw-scale medians from the training data. Both models receive separate indicators for missing WBC and glucose. The joint-category model has an unfavorable AUROC difference of −0.030 relative to the log-pair model; this result is included. The uncertainty does not establish equivalence. The machine-readable results contain full sensitivity-model coefficients, AP, Brier, scaled Brier, calibration intervals, and tuning results. AP, average precision; AUROC, area under the receiver operating characteristic curve; CI, confidence interval.

# Table S10. Multiplicative statistical interaction

| **Cohort** | **N/deaths** | **Interaction OR (Wald 95% CI)** | **LR P** | **Wald P** |
| --- | --- | --- | --- | --- |
| MIMIC-IV | 1589/324 | 1.00 (0.58–1.74) | 0.989 | 0.989 |
| eICU | 221/37 | 0.37 (0.06–2.28) | 0.279 | 0.283 |

The product term is I(WBC>10×10⁹/L)×I(glucose>140 mg/dL). Models retain the two binary main effects and the original cohort-specific clinical adjustment variables. The four-group factor and the two indicators with their product yield identical fitted probabilities: they are different parameterizations of the same model, not independent validations. MIMIC-IV uses ordinary LR tests, and eICU uses full-design penalized LR tests. The interaction concerns multiplicative odds, not additive risk.

eICU profile-penalized interaction CI: 0.058–2.251; P=0.279. The absence of clear statistical interaction does not prove absence of biological synergy. OR, odds ratio; CI, confidence interval; LR, likelihood ratio; WBC, white blood cell count.

## Supplementary computational checks

The archived regression analyses reproduced all six category comparisons to the precision reported in the submitted manuscript. Firth estimates were checked against an analytical saturated 2×2 example and equivalent categorical and product-term models. The spline analyses compared truncated-power and independently parameterized natural-spline bases, with fitted probabilities agreeing to better than 10⁻⁷. The prediction analysis recorded 53 passing checks covering metrics with tied values, calibration, training-only transformations, and finite probabilities. These checks assess the downstream implementation, not the accuracy of original event data. The integrated documents use the archived aggregate results and do not represent a repeat of every statistical fit.

# Table S11. Historical-to-revised prediction reconciliation

This table documents changes between the submitted and rebuilt prediction analyses; it is not an additional test of model superiority. “Original” refers to Table 4 in the submitted PDF. “Revised” refers to the explicitly specified prediction workflow used throughout the revised manuscript.

| **Model** | **Original/revised AUROC** | **Original/revised AP** | **Original/revised Brier** |
| --- | --- | --- | --- |
| C0 | 0.819 / 0.796 | 0.467 / 0.473 | 0.118 / 0.1178 |
| C_wbc | 0.826 / 0.818 | 0.485 / 0.500 | 0.118 / 0.1154 |
| C_glucose | 0.827 / 0.807 | 0.450 / 0.455 | 0.120 / 0.1185 |
| C_log | 0.828 / 0.818 | 0.454 / 0.458 | 0.118 / 0.1168 |
| C_rcs | 0.837 / 0.810 | 0.468 / 0.458 | 0.117 / 0.1177 |
| C_cat | 0.831 / 0.811 | 0.478 / 0.470 | 0.118 / 0.1162 |

The archived round11 script did not implement all ridge-regression and nested-validation procedures described in the submitted manuscript, and the historical predictive spline was incompletely documented. The rebuilt analysis specifies raw-marker imputation, scaling of continuous features, unscaled binary indicators, symmetric four-indicator category coding, training-only knot estimation, AP-based penalty selection, and shared validation splits. A fully specified predictive RCS replaces the historical B-spline.

Without the exact historical implementation and saved predictions, the cause of every numerical difference cannot be established. The differences are not attributed merely to rounding or software variation. Revised Tables 4/5, prediction figures, the abstract, and the related text consistently replace the earlier prediction values. The independently reproduced primary association ORs remain unchanged. Original AUPRC values are labeled AP here to reflect the intended average-precision summary; this relabeling does not verify the historical code.

Author names and affiliations follow the author-confirmed revised title page. All authors have approved the revised manuscript. These administrative updates do not alter the statistical results.

# Supplementary Figures S1–S2

Figure S1. Adjusted glucose–mortality curve from the joint three-knot Firth model in eICU (221 patients/37 deaths). The model includes age, sex, minimum GCS, SBP, and the WBC spline. The reference is glucose=140 mg/dL. Bands are pointwise Wald 95% CIs, and the display is limited to observed values. Only two patients had glucose <70 mg/dL; neither died.

Figure S2. Adjusted WBC–mortality curve from the same eICU Firth model, with WBC=10×10⁹/L as the reference. No patient had WBC <4×10⁹/L, so risk in the leukopenic range cannot be assessed. The curve and pointwise confidence bands are conditional on this model specification.

Abbreviations: GCS, Glasgow Coma Scale; WBC, white blood cell count; SBP, systolic blood pressure; CI, confidence interval; OR, odds ratio; AUROC, area under the receiver operating characteristic curve; RCS, restricted cubic spline.

# Supplementary Figures S3–S4

Figure S3. Full observed glucose range in the primary MIMIC-IV three-knot association model. All observations were also included in the model shown in main Figure 2. The display illustrates uncertainty at extreme values and does not identify a glucose treatment target.

Figure S4. Full observed WBC range in the primary MIMIC-IV model, including the maximum of 290.7×10⁹/L. These observations were retained. A targeted review of MIMIC-IV source-event tables identified repeated WBC measurements of the same order of magnitude in K/µL, although the submitted analytic extract does not retain row-level linkage to a specific source event. The central 98% analysis is a separate assessment of tail influence.

Abbreviations: GCS, Glasgow Coma Scale; WBC, white blood cell count; SBP, systolic blood pressure; CI, confidence interval; OR, odds ratio; AUROC, area under the receiver operating characteristic curve; RCS, restricted cubic spline.

# Supplementary Figures S5–S6

Figure S5. Glucose–mortality curve from the four-knot MIMIC-IV sensitivity model using all patients. The model also includes a four-knot WBC spline and the original clinical adjustment variables. Bands show approximate pointwise Wald CIs.

Figure S6. WBC–mortality curve from the four-knot MIMIC-IV sensitivity model using all patients. Its fitted curve changes direction and differs from the primary three-knot curve. This result is reported alongside, rather than instead of, the primary analysis.

Abbreviations: GCS, Glasgow Coma Scale; WBC, white blood cell count; SBP, systolic blood pressure; CI, confidence interval; OR, odds ratio; AUROC, area under the receiver operating characteristic curve; RCS, restricted cubic spline.

# Supplementary Figures S7–S8

Figure S7. Glucose–mortality curve in the central 98% sensitivity analysis. Records outside either marker's original primary-sample 1st–99th percentile interval were excluded, leaving 1,528 patients/304 deaths. Original knot locations, reference values, and clinical adjustment were retained; glucose nonlinearity P=0.024.

Figure S8. WBC–mortality curve in the same central 98% sensitivity analysis. Exclusion was based on the two observed marker distributions, not the outcome or an assumed diagnosis. WBC overall P<0.001 and nonlinearity P=0.222; the primary analysis sample is unchanged.

Abbreviations: GCS, Glasgow Coma Scale; WBC, white blood cell count; SBP, systolic blood pressure; CI, confidence interval; OR, odds ratio; AUROC, area under the receiver operating characteristic curve; RCS, restricted cubic spline.

# Supplementary Figures S9–S10

Figure S9. Glucose point-estimate curves from all three specified MIMIC-IV models: the primary three-knot model, the four-knot model, and the central 98% three-knot sensitivity model. The broad increasing pattern and curvature are more consistent than for WBC, but low-glucose observations remain sparse.

Figure S10. Paired Brier-score changes in eICU for extended prediction models versus their corresponding baselines. Lower values favor the extended model. Intervals use the same 2,000 patient-level bootstrap samples with predictions held fixed. Both principal joint-category comparison intervals span zero.

Abbreviations: GCS, Glasgow Coma Scale; WBC, white blood cell count; SBP, systolic blood pressure; CI, confidence interval; OR, odds ratio; AUROC, area under the receiver operating characteristic curve; RCS, restricted cubic spline.

# Supplementary Figures S11–S12

Figure S11. Grouped calibration in eICU for GCS alone and GCS with the joint category added. Wilson intervals describe observed mortality within groups defined by predicted-probability quantiles, not uncertainty bands for the entire calibration function. Predictions were not recalibrated.

Figure S12. Receiver operating characteristic curves for the clinical baseline model and its marker extensions in the same eICU sample. These discrimination curves alone do not establish the shapes of individual marker associations or their additional clinical usefulness.

Abbreviations: GCS, Glasgow Coma Scale; WBC, white blood cell count; SBP, systolic blood pressure; CI, confidence interval; OR, odds ratio; AUROC, area under the receiver operating characteristic curve; RCS, restricted cubic spline.
